# ﻿A high species diversity of *Lyomyces* (Basidiomycota, Hymenochaetales) in Central and South America, revealed after morphological and molecular analysis

**DOI:** 10.3897/mycokeys.109.127606

**Published:** 2024-10-03

**Authors:** Eugene Yurchenko, Ewald Langer, Janett Riebesehl

**Affiliations:** 1 Institute of Forest Sciences, Białystok University of Technology, Wiejska 45A, 15-351, Białystok, Poland Białystok University of Technology Białystok Poland; 2 Department of Ecology, University of Kassel, Heinrich-Plett-Str. 40, DE-34132, Kassel, Germany University of Kassel Kassel Germany; 3 Institute for Plant Protection in Horticulture and Urban Green, Julius Kühn Institute, Messeweg 11/12, DE-38104, Braunschweig, Germany Institute for Plant Protection in Horticulture and Urban Green, Julius Kühn Institute Braunschweig Germany

**Keywords:** Crystals SEM, montane forests, Neotropics, taxonomy, 28S rDNA

## Abstract

A study of corticioid fungi collections from Costa Rica, Panama, Colombia, and Ecuador has resulted in the identification of 26 morphospecies of *Lyomyces*. These distinctions were made based on characters such as basidiospore size and shape, cystidia morphology, basidioma texture, and hymenial surface configuration. Sequences of rDNA ITS were obtained for 12 of these species, and their relationships to previously known taxa were illustrated using Bayesian and Maximum Likelihood reconstructions of the phylogeny. Protologues are given for 10 new species of *Lyomyces*: *L.boquetensis* (found in Panama, belonging to *L.sambuci* group), *L.granulosus* (from Costa Rica and Panama, related to *L.fimbriatus*), *L.napoensis* (from Ecuador, related to *L.elaeidicola*), *L.neocrustosus* (from Panama, *L.crustosus* group), *L.oleifer* (from Ecuador, *L.crustosus* group), *L.pantropicus* (from Panama and Ecuador, related to *L.microfasciculatus*), *L.orarius* (from Ecuador, *L.sambuci* group), *L.parvus* (from Costa Rica and Ecuador, *L.crustosus* group), *L.sceptrifer* (from Ecuador, related to *L.gatesiae*), and *L.subcylindricus* (from Panama, *L.crustosus* group). Macro- and micro-morphology illustrations are provided for these new species. Additionally, the range of *L.organensis* is extended to Ecuador. Scanning electron microscopy of crystalline deposits in basidiomata has shown the differences in crystal size and aggregation manner between species.

## ﻿Introduction

The genus *Lyomyces* (Schizoporaceae, Hymenochaetales, Agaricomycetes) was initially described by Karsten (1881), who designated *L.serus* (Pers.) P. Karst. as the type species, known under the current name *L.sambuci* (Pers.) P. Karst. After publishing a new combination *Hyphodontiasambuci* (Pers.) J. Erikss. for the type species (Eriksson 1958), *Lyomyces* was referred for a considerable period to the synonyms of *Hyphodontia* J. Erikss. However, in the work by Hjortstam and Ryvarden (2009), the genus *Lyomyces* was reused and distinguished from the closest related genus *Xylodon* (Pers.) Gray. The separation from *Xylodon* was also supported by molecular phylogeny (Riebesehl and Langer 2017). Nevertheless, the support for the *Lyomyces* clade is not consistently strong in phylograms (Yurchenko et al. 2017, 2020). Furthermore, the small genus *Rogersella* Liberta & A.J. Navas was included in *Lyomyces* following the arguments from molecular phylogeny (Riebesehl and Langer 2017).

*Lyomyces* comprises corticioid fungi characterised by thin, effused, membranaceous basidiomata that appear fragile in a dry state. The hymenial surface is predominantly white or whitish, and the hyphal system is monomitic. The subicular hyphae are thin- or somewhat thick-walled, while the cystidia are thin-walled with tapering, cylindrical, subcapitate, or capitate apical parts. The basidia are utriform, and the basidiospores are colourless with thin to thick, smooth, or occasionally minutely warted walls. These characteristics, with the exception of warted spores, align well with the generic type, *L.sambuci*. The configuration of the hymenial surface can vary from smooth to densely odontoid. In some mature basidiomata, cream or pale ochraceous pigmentation can accumulate. Crystalline deposits on hyphae and hymenial elements are a distinguishing feature of most *Lyomyces* species, and at later stages of basidioma development, crystalline masses can predominate in their texture.

Although the morphological differences between *Lyomyces* and *Xylodon* are not entirely distinct, the pigmentation of basidioma elements serves as a delimiting character between these genera. In *Lyomyces*, the hyphae are colourless, and the hymenial surface is white or yellowish, seldom pale ochraceous. On the other hand, in *Xylodon*, the subicular and tramal hyphae are usually pale-coloured in mass, and the hymenial surface often exhibits brownish-yellow hues.

The species of *Lyomyces* are saprobes that inhabit the decaying wood and bark of both angiosperms and gymnosperms. Occasionally, they can be found on dead herbaceous stems such as *L.capitatocystidiatus* (H.X. Xiong, Y.C. Dai & Sheng H. Wu) Riebesehl & Langer (Xiong et al. 2009), and on dead parts of ferns like *L.griseliniae* (Langer 1994). In rare cases, there have been observations of basidiomata on growing parts of trees and bushes (Yurchenko 2008; Dreistadt 2012; Wang et al. 2021), suggesting a potential endophytic lifestyle for at least one species, *L.sambuci*. Additionally, a number of *Lyomyces* species display a notable tolerance to drying and can be found on dead but still attached branches.

This paper presents the taxonomic results of sampling *Lyomyces* from four countries in Central and South America: Costa Rica, Panama, Colombia, and Ecuador. As part of this study, ten new species are described as new for science, contributing to the limited number of previously published records of *Lyomyces* from this region. Prior to our research, in Colombia, the recorded species included *Lyomyces* cfr. *crustosus* (as *Hyphodontia* cfr. *crustosa* (Pers.) J. Erikss.), *L.juniperi* (as *H.juniperi* (Bourdot & Galzin) J. Erikss.), *L.stratosus* (as *H.stratosa* Hjortstam & Ryvarden; Hjortstam and Ryvarden 1997, 2007b), and *L.griseliniae* (as *Rogersellagriseliniae* (G. Cunn.) Stalpers; Hjortstam and Ryvarden 2001, 2007b). The most recent checklist of Colombian fungi (de Almeida et al. 2022) includes three *Lyomyces* species: *L.crustosus*, *L.griseliniae*, and *L.juniperi*. As for the other countries in the study region, no *Lyomyces* records were previously documented, despite some fragmentary studies of corticioid fungi conducted in Costa Rica (Kisimova-Horovitz et al. 1997; Hjortstam and Ryvarden 2007a), Panama (Piepenbring 2006), and Ecuador (Hjortstam and Ryvarden 2008).

## ﻿Materials and methods

### ﻿Collections and studies of morphology

The specimens were collected during trips conducted by E. Langer to the central part of Costa Rica (1989), and to southern Ecuador (2001, 2002, 2004), and by E. Yurchenko to South and Central America (2019). The field studies by E. Yurchenko were carried out in the northern part of Ecuador (Esmeraldas Province, natural region of Costa; Pichincha Province, natural region of Andes), the northeast part of Ecuador (Orellana Province, natural region of Amazonia), and in the western part of Panama. Furthermore, specimens collected in the northern and central parts of Colombia by L. Ryvarden in 1978 were critically examined, along with some corticioid fungi collected in central Costa Rica by L. Kisimova-Horovitz between 1991 and 1997.

Fungi were collected from dead wood, stems, and litter components, ranging from ground level to approximately 1.5–2 metres above the ground. The collection was carried out using the standard route-based method and, in certain cases, with more detailed sampling on permanent and temporary plots within forest areas. Field studies covered ecosystems which were affected differently by human activity, ranging from pristine mountain forests to areas near roadsides and agricultural lands. Examined specimens are deposited in herbaria BLS, CFMR, KAS, and O (the acronyms follow Index Herbariorum, http://sweetgum.nybg.org/science/ih).

Dry specimens were used for descriptions and illustrations. The thickness of basidiomata was also measured on the dry specimens. Micromorphology was studied using slides prepared in a 3% aqueous solution of KOH (abbreviated as KOH in the text). Additionally, crystalline incrustations and spores were examined in Melzer’s reagent (**Mz**). The absence of an amyloid or dextrinoid reaction of the spore wall is abbreviated as **Mz**– in the text. To assess the cyanophily of the spores, 0.02% cotton blue in 50% aqueous lactic acid was used. Microstructure measurements were conducted using a Nikon Eclipse Ni-U light microscope (Nikon Corp., Japan) equipped with NIS-Elements Br imaging software (Nikon Corp.), mostly under ×1000 magnification. Mean spore length (**L**) and width (**W**) were calculated as arithmetic averages from measurements of 30 randomly selected spores in squash preparations or vertical sections of basidiomata. The spore quotient (**Q**) was obtained by calculating the L/W ratio for each individual spore. Spores observed under magnification ×1000 were classified as follows: thin-walled—if their wall appeared as one thin layer, slightly thick-walled—if two unclear layers were observed, and thick-walled—if the wall appeared as a double line (Wu 1990). According to other definitions, slightly thickened spore walls do not exceed 0.5 μm, whereas thick walls are more than 0.5 μm in thickness (Maekawa et al. 2023).

Scanned electron (SEM) images of crystalline deposits were captured using a Phenom G2 pro desktop microscope (Labmate, UK). To achieve this, pieces of basidiomata measuring 3–4 mm in size were taken from the herbarium and affixed to aluminium pin stubs using double-sided carbon adhesive discs (Leit tabs). A thin layer of gold, approximately 5.2 nm thick, was then applied to the samples using a Leica EN ACE200 vacuum coater (Leica Microsystems, Germany). Finally, the samples were examined at various magnifications, ranging from ×5000 to ×17000.

### ﻿DNA extraction, sequencing, and phylogenetic analysis

For DNA preparation, pieces of dry basidiomata, 15–50 mm^2^ in size, were cut without substratum, or with as small an amount of substratum as possible. DNA extraction was done with an E.Z.N.A.^®^ Fungal DNA Mini Kit (Omega Bio-tek, Georgia, USA). Primers ITS1, ITS2, ITS3, ITS4, ITS5 (White et al. 1990), ITS1F (Gardes and Bruns 1993), 58AF1 (Martin and Rygiewicz 2005), ITS2.2fXyl, ITS2.2rXyl (Viner et al. 2021), ALR0.2 (Riebesehl and Langer 2017), and LR22 (https://sites.duke.edu/vilgalyslab/files/2017/08/rDNA-primers-for-fungi.pdf) were used for DNA amplification of ribosomal internal transcribed spacer (ITS). Two reads were obtained for the majority of specimens: ITS1+partial 5.8S gene and partial 5.8S gene+ITS2. The beginning D1-D2 domains of the 28S rRNA gene were amplified with primers LR5 (Vilgalys and Hester 1990), LR0R (Bunyard et al. 1996), and NL1 and NL4 (O’Donnell 1993). Purifications of the PCR products were done with a DNA Clean & Concentrator^®^-5 kit (Zymo Research, Irvine, California, USA) and DNA sequencing was implemented by LGC Genomics GmbH (Berlin, Germany). The new sequences were edited and assembled in MEGA 11 (Tamura et al. 2021), under the consideration of five quality check guidelines (Nilsson et al. 2012) and were then deposited in the NCBI GenBank (https://www.ncbi.nlm.nih.gov/genbank; Table 1; see also Results).

**Table 1.** ITS and 28S sequences, used in phylogenetic reconstructions.^†^

Species	Specimen / voucher code	GenBank accession No.		Reference
		ITS	28S	
*Hyphodontia* sp.	‘sp. 01’ RLC_582_iNat_122493200	OQ871786		Vandegrift et al. (2023)
*Lyomycesalbopulverulentus* C.L. Zhao	CLZhao 21478 (SWFC), holotype	OP730712	OP730724	Guan et al. (2023)
*L.allantosporus* Riebesehl, Yurchenko & Langer	FR-0249548, holotype	KY800397; NR_154135	KY795963	Yurchenko et al. (2017)
*L.austro-occidentalis* Xue W. Wang & L.W. Zhou	CLZhao 9819 (SWFC), as holotype of *L.ochraceoalbus* C.L. Zhao	MZ262538	MZ262524	Luo et al. (2021b)
*L.bambusinus* C.L. Zhao	CLZhao 4831 (SWFC), holotype	MN945968; NR_176132	MW264919; NG_153936	Chen and Zhao (2020)
***L.boquetensis* Yurchenko & Riebesehl**	**EYu 190727-12 / BLS M-5238, holotype**	**PP471797**	**PP471818**	**this study**
*L.cremeus* C.L. Zhao	CLZhao 8295 (SWFC), holotype	MN945972		Chen and Zhao (2020)
*L.crustosus* (Pers.) P. Karst.	KAS-GEL2325	DQ340313		this study
	KAS-GEL5360	DQ340315		this study
	Spirin 12630 (H)	OK273832	OK273832	Viner et al. (2021)
*L.crystallina* Xue W. Wang & L.W. Zhou	LWZ 20190810-6b (HMAS), holotype	OQ540901		Liu et al. (2024)
*L.densiusculus* Viner & Ryvarden	LR 44818 (O), holotype	OK273853	OK273853	Viner et al. (2021)
*L.denudatus* Viner	LR 19436 / H 7006820, holotype	ON980760		Viner and Miettinen (2022)
*L.elaeidicola* Xue W. Wang & L.W. Zhou	LWZ 20180411-20 (HMAS), holotype	MT319458; NR_182827	MT319192; NG_153910	Wang et al. (2021)
*L.erastii* (Saaren. & Kotir.) Hjortstam & Ryvarden	LWZ 20160907-5 (IFP)	MT319453	MT319188	Wang et al. (2021)
*L.fimbriatus* (Sheng H. Wu) Riebesehl & Yurchenko	Wu 911204-4 / CFMR 37006	MK575210	MK598740	Yurchenko et al. (2020)
*L.fissuratus* C.L. Zhao	CLZhao 4291 (SWFC), holotype	MW713738; NR_189834	MW713730; NG_242493	Luo et al. (2021a)
*L.fumosus* C.L. Zhao	CLZhao 8188 (SWFC), holotype	MW713744; NR_189835	MW713736; NG_242494	Luo et al. (2021a)
*L.gatesiae* Xue W. Wang & L.W. Zhou	LWZ 20180515-3 (MEL), holotype	MT319447; NR_182826	MT319181; NG_153909	Wang et al. (2021)
***L.granulosus* Yurchenko & Langer**	**KAS-GEL1662, holotype**	**PP471799**		**this study**
***L.granulosus***	**EYu 190727-8b / BLS M-2975**	**PP471798**	**PP471819**	**this study**
*L.griseliniae* (G. Cunn.) Riebesehl & Langer	KHL 12971 (GB)	DQ873651	OK273851	Larsson et al. (2006)
*L.guttulatus* Xue W. Wang & L.W. Zhou	LWZ 20200921-29a (HMAS), holotype	OQ540899	OQ540859	Liu et al. (2024)
*L.incrustatus* (Kotir. & Saaren.) Hjortstam & Ryvarden	Viner 2019_203 (H)	ON197553	ON197553	Viner et al. (2023)
*L.lancangjiangensis* Qi Li & C.L. Zhao	CLZhao 25338 (SWFC), holotype	OR844490	OR891518	Li et al. (2024)
*L.leptocystidiatus* Xue W. Wang & L.W. Zhou	LWZ 20170814-14 (IFP), holotype	MT319429; NR_182825	MT319163; NG_153908	Wang et al. (2021)
*L.macrosporus* C.L. Zhao	CLZhao 8605 (SWFC), holotype	MN945975		Chen and Zhao (2020)
*L.mascarensis* Riebesehl, Yurchenko & Langer	KAS-GEL4833	KY800399; NR_154136	KY795964; NG_060351	Yurchenko et al. (2017)
*L.microfasciculatus* (Yurchenko & Sheng H. Wu) Riebesehl & Langer	Wu 910808-56 / TNM F24757, holotype	JN129976		Yurchenko and Wu (2014)
*L.microfasciculatus*	He 5710 (BJFC)	MW507091		unpublished
*L.microfasciculatus*	CLZhao 3634 (SWFC)	MK269032		unpublished
*L.microfasciculatus*	CLZhao 4574 (SWFC)	OM959416; MK343567		unpublished
*L.microfasciculatus*	CLZhao 16067 (SWFC)	MW578327		unpublished
*L.microfasciculatus*	LWZ 20170815-37 (IFP)	MT319450	MT319184	Wang et al. (2021)
***L.napoensis* Yurchenko & Riebesehl**	**EYu 190720-18 / BLS M-2610, holotype**	**PP471800**	**PP471820**	**this study**
***L.neocrustosus* Yurchenko & Riebesehl**	**EYu 190728-14 / BLS M-5239, holotype**	**PP471801**	**PP471821**	**this study**
*L.niveus* C.L. Zhao ex L.W. Zhou & Xue W. Wang	CLZhao 6496 (SWFC), holotype	MZ262545	MZ262530	Luo et al. (2021b)
***L.oleifer* Yurchenko & Langer**	**KAS-Ec47-2001, holotype**	**PP471802**		**this study**
	**KAS-Ec287-2001**	**PP471803**		**this study**
	**KAS-Ec423-2001**	**PP471804**		**this study**
***L.orarius* Yurchenko & Riebesehl**	**EYu 190724-1 / BLS M-2995, holotype**	**PP471805**	**PP471822**	**this study**
*L.organensis* Yurchenko & Riebesehl	MSK-F 7247, holotype	KY800403	KY795967	Yurchenko et al. (2017)
***L.organensis***	**KAS-Ec180-2004**	**PP471806**	**PP471823**	**this study**
*L.orientalis* Riebesehl, Yurchenko & Langer	KAS-GEL3400	DQ340326	DQ340353	Yurchenko et al. (2017)
***L.pantropicus* Yurchenko & Riebesehl**	**EYu 190727-23b / BLS M-2679, holotype**	**PP471808**	**PP471825**	**this study**
***L.pantropicus***	**EYu 190724-12 / BLS M-3447**	**PP471807**	**PP471824**	**this study**
***L.parvus* Yurchenko & Langer**	**KAS-GEL1599, holotype**	**PP471810**		**this study**
***L.parvus***	**EYu 190718-23 / BLS M-2990**	**PP471809**	**PP471826**	**this study**
*L.pruni* (Lasch) Riebesehl & Langer	KAS-GEL2327	DQ340312	DQ340349	Yurchenko et al. (2020)
*L.punctatomarginatus* Qi Li & C.L. Zhao	CLZhao 11629 (SWFC), holotype	OR844491	OR891519	Li et al. (2024)
*L.sambuci* (Pers.) P. Karst.	KAS-JR7	KY800402	KY795966	Yurchenko et al. (2017)
	KAS-GEL2414	KY800398		Yurchenko et al. (2017)
***L.sceptrifer* Yurchenko & Langer**	**KAS-Ec661-2002, holotype**	**PP471811**	**PP471827**	**this study**
***L.subcylindricus* Yurchenko & Riebesehl**	**EYu 190727-25 / BLS M-2668, holotype**	**PP471817**		**this study**
	**EYu 190727-10a / BLS M-2992**	**PP471816**	**PP471832**	**this study**
*L.tasmanicus* Xue W. Wang & L.W. Zhou	LWZ 20180515-17 (HMAS), holotype	OQ540900	OQ540860	Liu et al. (2024)
*L.vietnamensis* (Yurchenko & Sheng H. Wu) Riebesehl & Langer	Wu 9807-88 / TNM F9073, holotype	JX175044	KX857814	Yurchenko and Wu (2014)
*L.wuliangshanensis* C.L. Zhao	CLZhao 4167 (SWFC), holotype	MN945979		Chen and Zhao (2020)
*L.yunnanensis* C.L. Zhao	CLZhao 10041 (SWFC), holotype	OP730709		Guan et al. (2023)
*Lyomyces* sp.	LWZ 20180512-6 (IFP)	MT319446		Wang et al. (2021)
*Lyomyces* sp. 3	97SAMHYP / LR 19785 (O)	JX857746		unpublished
*Xylodonquercinus* (Pers.) Gray	Spirin 12030 (H)	OK273841	OK273841	Viner et al. (2021)
*Xylodonstratosus* (Hjortstam & Ryvarden) Hjortstam & Ryvarden	LR 9798 (O), holotype	KY081805		Riebesehl and Langer (2017)
	LR 10025 (O)	KY081806		Riebesehl and Langer (2017)

^†^ Sequences obtained in this study are given in bold.

The ITS and 28S datasets for phylogenetic analyses included newly generated sequences and sequences of *Lyomyces* available from NCBI GenBank (Table 1). At least one representative sequence of each available *Lyomyces* species was selected. *Xylodonquercinus*, the generic type of *Xylodon*, was assigned as the outgroup. The alignment was done with MAFFT v. 7.511 (Katoh et al. 2019; https://mafft.cbrc.jp/alignment/server) using the L-INS-i algorithm for ITS and G-INS-i for the 28S dataset. The aligned matrices were edited in MEGA 11 (Suppl. materials 1, 2). The Maximum Likelihood (ML) phylograms were computed in MEGA 11 under the usage of a substitution model based on BIC and suggested by the MEGA 11 Model Selection tool. Bootstrap replications of 1000, and other settings as default were applied. Bayesian inference (BI) phylograms were computed with TrEase (Mishra et al. 2023; http://www.thines-lab.senckenberg.de/trease) using MrBayes v. 3.2 (Ronquist et al. 2012) under the usage of the same substitution model, one million generations, with a burn-in of 25% of trees to discard, and other parameters as default. FigTree v. 1.4.4 (http://tree.bio.ed.ac.uk/software/figtree) was used for processing the BI phylograms. The ready phylograms were prepared in CorelDRAW v. 9 (Corel Corp., Ottawa, Canada, 1999).

## ﻿Results

### ﻿Phylogenetic inference

The ITS rDNA barcodes were derived for 20 specimens of *Lyomyces* from the study area. The aligned ITS data matrix consisted of 64 taxa and 738 positions. It was partitioned as follows: ITS1 = positions 1–288, 5.8S rRNA gene = 289–467, and ITS2 = 468–738. The best model suggested by the Model Selection tool in MEGA 11 was GTR+G.

28S rDNA barcodes were derived for 14 specimens. The combined data matrix of ITS and 28S sequences consisted of 39 taxa and 1362 positions. It was partitioned as follows: ITS = 1–738 and 28S = 739–1362. The best model suggested by the Model Selection tool in MEGA 11 was GTR+G+I.

**Figure 1.**
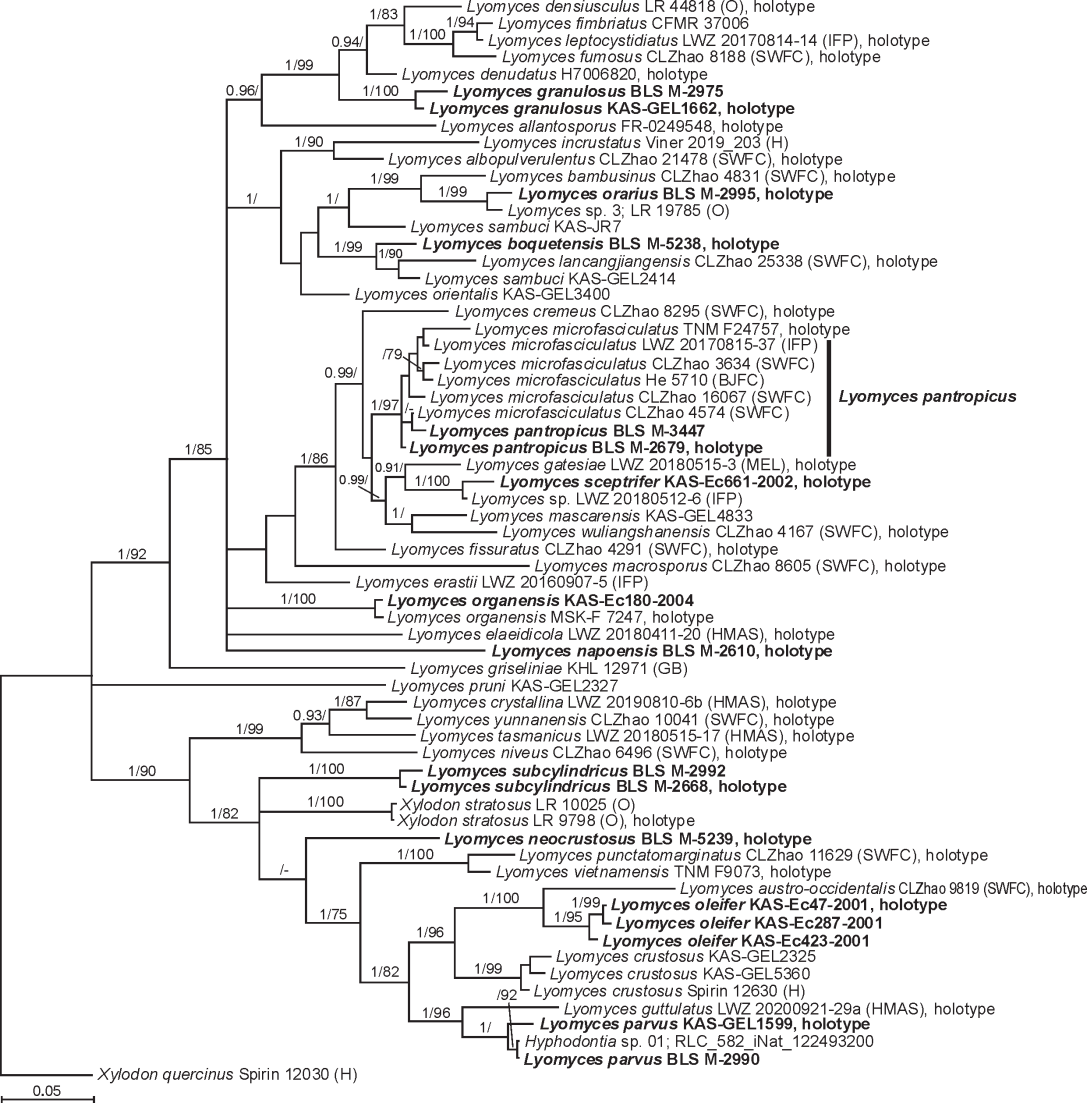
Bayesian reconstruction of the phylogeny for the *Lyomyces* species based on ITS sequences. Numbers above branches denote posterior probability values (PP ≥ 0.9) / ML bootstrap values (BS ≥ 75%) from the ML phylogram; ‘-’ denotes the absence of the branch in the ML phylogram. Sequences obtained in this study are shown in bold. Scale bar: number of substitutions per site.

**Figure 2.**
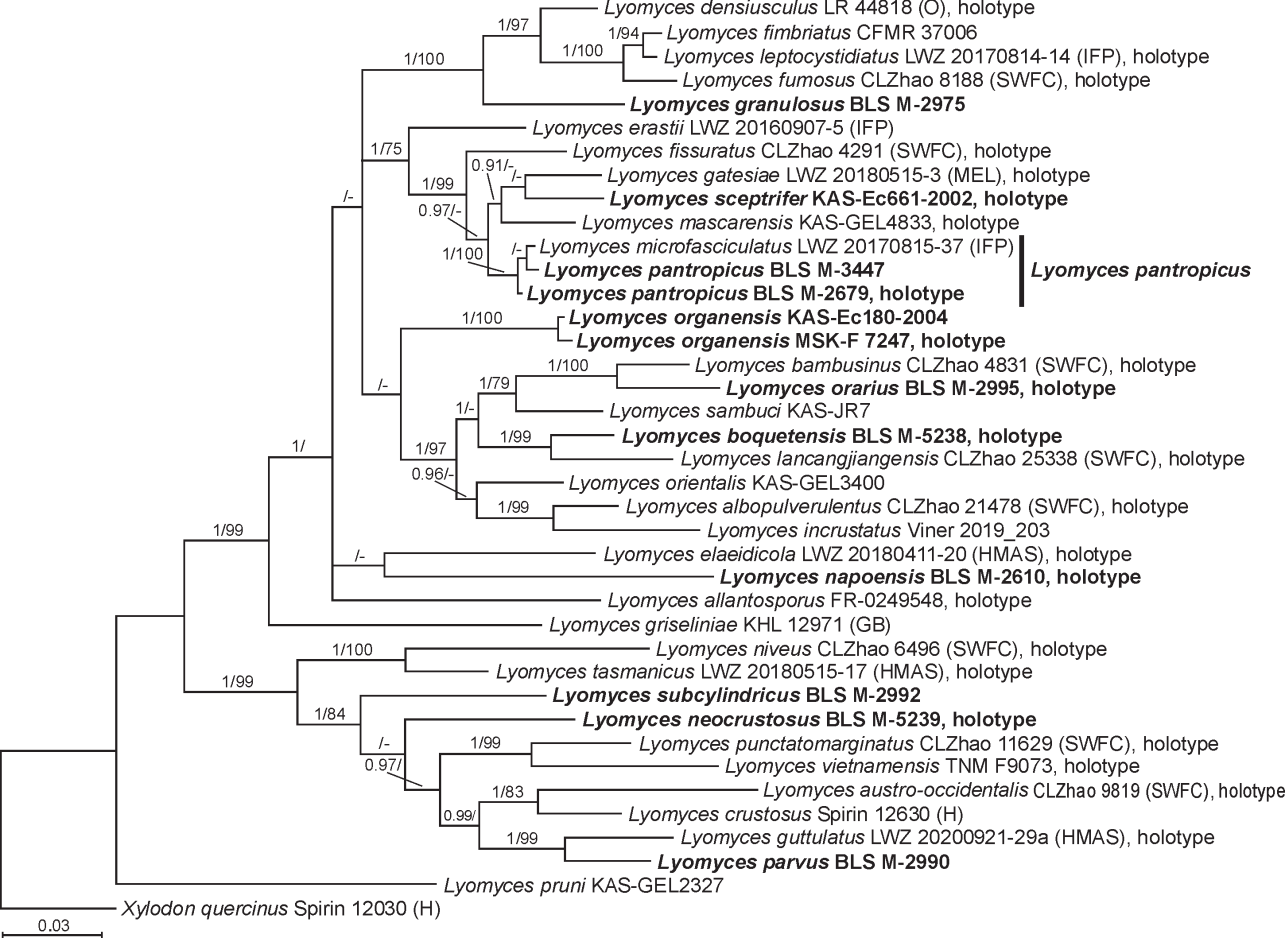
Bayesian reconstruction of the phylogeny for the *Lyomyces* species based on a combined ITS+28S dataset. Numbers above branches denote posterior probability values (PP ≥ 0.9) / ML bootstrap values (BS ≥ 75%) from the ML phylogram; ‘-’ denotes the absence of the branch in the ML phylogram. Sequences obtained in this study are shown in bold. Scale bar: number of substitutions per site.

The phylogenetic reconstructions (Figs 1, 2) confirmed that 17 specimens, from which ITS barcodes were derived, belong to the genus *Lyomyces*. The ML phylograms demonstrated highly similar topology with the reconstructions based on BI. The inference of the phylogeny demonstrated that our sequences from specimens named *L.boquetensis*, *L.granulosus*, *L.napoensis*, *L.neocrustosus*, *L.oleifer*, *L.orarius*, *L.parvus*, *L.sceptrifer*, and *L.subcylindricus* represent independent species, based on branch length and their positions among the previously known species.

We found that ITS sequence OQ871786 from GenBank, derived from the specimen *Hyphodontia* sp. 01 RLC_582_iNat_122493200, collected in cloud forest in the Andes Mountains, northwest Ecuador (Vandegrift et al. 2023), was 99.53% similar with the ITS of *L.parvus* holotype, which is also seen at the phylogram (Fig. 1). ITS sequence MT319446 derived from specimen LWZ 20180512-6, collected in Australia, occurred to be 97.37% similar to *L.sceptrifer* holotype, with 87% of alignment coverage. Another fungal specimen from South Vietnam (GenBank: ITS = MF942542) demonstrated 98.54% similarity with *L.sceptrifer*, but with only 73% coverage. Because of the low alignment coverage, we do not state the final attribution of the two latter sequences to *L.sceptrifer*.

The ITS sequences obtained from BLS M-2679 and BLS M-3447 showed a high similarity (99.47–100%) to sequences from China (GenBank MK269032, MW578327) and Sri Lanka (GenBank MW507091), identified as *L.microfasciculatus*. However, there is a comparatively low similarity (96.99%) between the holotype of *L.microfasciculatus* and the selected holotype of *L.pantropicus*, which gives grounds to consider the latter as a separate species.

The specimen KAS-Ec180-2004 showed a near-identical match (99.66% ITS similarity) with the holotype of *L.organensis*, indicating that they belong to the same species. Additionally, three of our specimens, BLS M-2994 from Panama (GenBank: ITS = PP471814, 28S = PP471830), BLS M-9933 from Panama (younger basidiomata patches ITS = PP471812, 28S = PP471828; older basidiomata patches ITS = PP471813, 28S = PP471829), and KAS-Ec199-2004 from Ecuador (ITS = PP471815; 28S = PP471831), were supposedly conspecific with five unpublished specimens from GenBank conventionally named *Lyomyces* sp. 1. After alignment, the pairwise distances with these specimens were less than 1%. Sequences of *Lyomyces* sp. 1 were deposited in 2012 by M.P. Martín, M. Dueñas, and M.T. Telleria, and originate from the Azores Islands (JX857739, JX857741, JX857742), Madeira Island (JX857743), and the Jujuy Province of Argentina (JX857740).

### ﻿Morphological analysis

After studying corticioid fungi collections from Costa Rica, Panama, Colombia, and Ecuador, a total of 47 specimens were identified as belonging to the genus *Lyomyces*. Out of these, DNA barcodes were obtained for 20 specimens. For the specimens without DNA barcodes, we relied on the generic morphological concept (as explained in the Introduction) to assign them to *Lyomyces*. Furthermore, we compared the morphology of these specimens with those that had ITS rDNA barcodes and whose position in *Lyomyces* was confirmed through phylogenetic reconstructions. This comparative approach helped us in making accurate identifications within the genus.

After evaluating all the main morphological features, we found that the diversity of *Lyomyces* species can be classified using the characters in the following hierarchy: 1) spore shape (Q); 2) spore size; 3) spore wall thickness; 4) hymenophore configuration and basidioma texture; 5) main types of cystidia and its frequency in hymenia. To organize the classification, we first grouped the species based on their spore shape at the primary level. Then, at the secondary level, we further grouped them based on their spore size range, and so on. By employing these method, we classified the specimens from the study area into 26 named and unnamed (taxonomic) species. Ten species whose morphology does not match any known taxa, are described below as new. Among them, *L.pantropicus* was distinguished from *L.microfasciculatus* mainly based on morphological traits.

We found that according to morphology, the specimens from Colombia stored in O under the name *L.crustosus* (O-F-918427, O-F-918428, O-F-918425) should belong to the three different species that are not identical to the European *L.crustosus*. One of them, O-F-918425, happened to be similar to a specimen from Panama BLS M-5239, described below as a new species, *L.neocrustosus*. The specimens from Colombia called *L.juniperi* on labels (O-F-918431, O-F-918432), are the two morphologically distinct species.

### ﻿Taxonomy

#### ﻿ Lyomyces boquetensis

Taxon classificationFungiCorticialesHymenochaetales

Yurchenko & Riebesehl sp. nov.

9C380374-AA31-5CB6-9093-3F80E6D8BF69

850097

**Figure 3.**
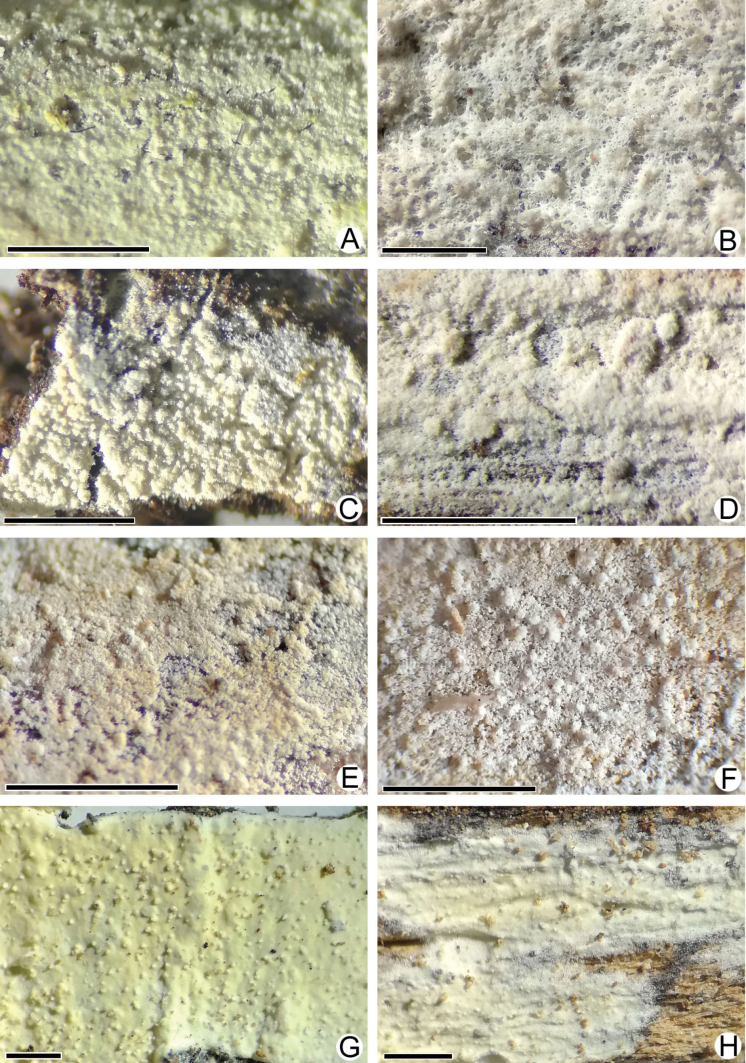
Macromorphology of *Lyomyces* species **A, B***L.boquetensis*, holotype (BLS M-5238) **C***L.granulosus*, holotype (KAS-GEL1662) **D***L.granulosus*, BLS M-2975 **E, F***L.napoensis*, holotype (BLS M-2610) **G, H***L.neocrustosus*, holotype (BLS M-5239). Scale bars: 1 mm.

**Figure 4.**
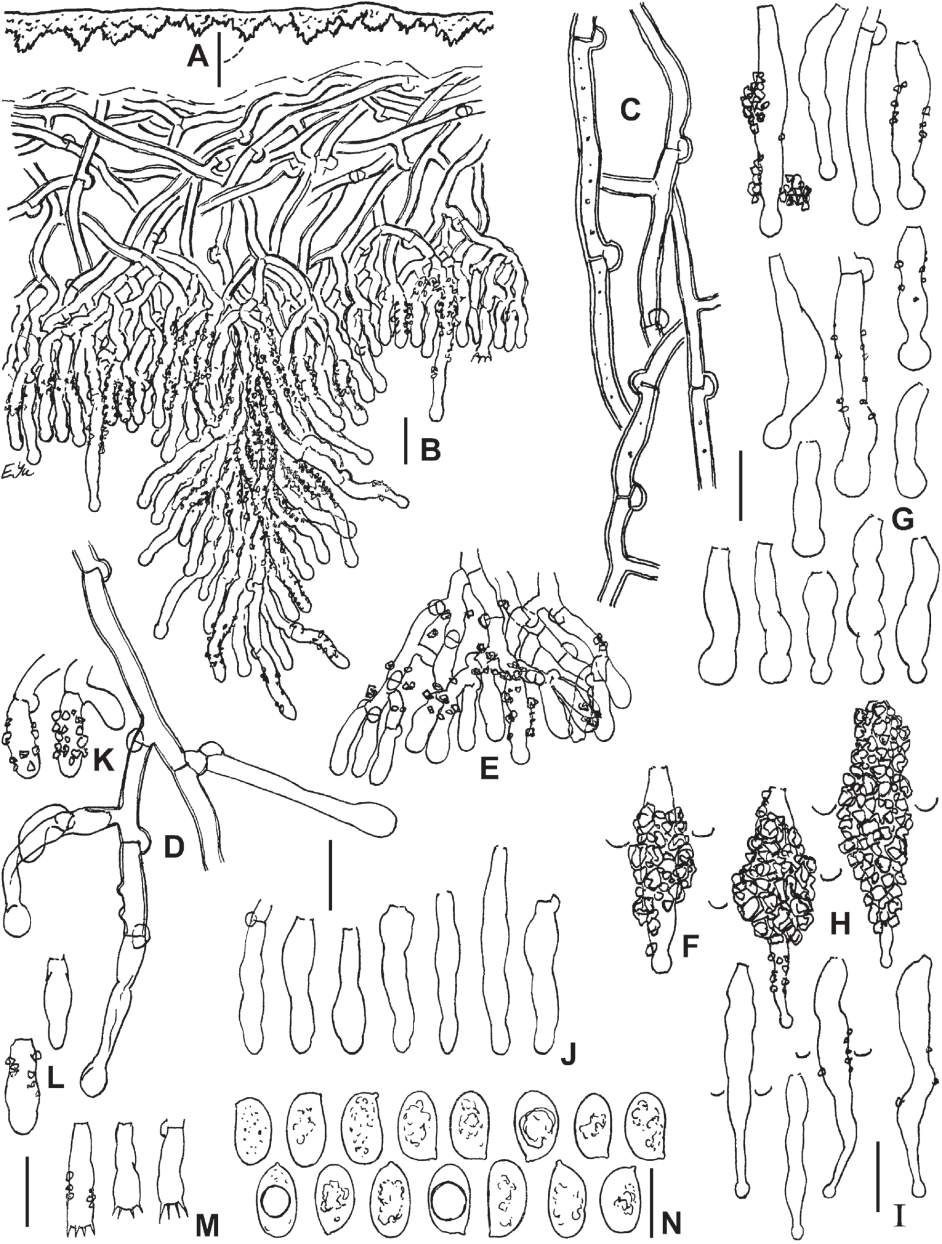
Micromorphology of *Lyomycesboquetensis* (holotype, BLS M-5238) **A, B** vertical sections through basidioma **C** subicular hyphae **D** hyphae from hymenophore wart, ended by capitate cystidia **E** portion of hymenium and subhymenium **F, G** capitate and subcapitate cystidia **H**, **I** capitulate cystidia **J** cylindrical cystidia **K, L** basidioles **M** basidia **N** basidiospores. **B–E, G, I, J, L–N** in KOH; **F, H, K** in Mz. Scale bars: 100 μm (**A**); 10 μm (**B–M**); 5 μm (**N**).

Figs 3A, B , 4 , 16A, B

##### Type.

Panama • Chiriquí Province: W of Boquete town, Bajo Boquete community, 08°46.58'N, 082°28.17'W, 1450 m a.s.l., evergreen montane tropical forest, bottom of canyon with a rivulet, on fallen corticated twig of angiosperm, 27 Jul 2019, E. Yurchenko EYu 190727-12 (***holotype***: BLS M-5238; ***isotype***: CFMR). GenBank: ITS = PP471797; 28S = PP471818.

##### Etymology.

*boquetensis* = refers to Boquete, the town near the type locality.

##### Description.

Basidiomata effused, 1.5–3 and more cm in extent, soft-membranaceous to membranaceous, from fine flocculose or granulose and discontinuous when young to continuous, 20–60 μm thick between warts. Hymenial surface white or dirty whitish, smooth to minutely warted; warts up to 3–7/mm, 15–60 μm high. Margin concolourous, appressed mould-like or diffuse, about 0.5 mm wide. Hyphal system monomitic, hyphae clamped at all septa, smooth (in KOH) or slightly encrusted (in Mz). Subiculum of loose texture; subicular hyphae moderately branched, 2–4 μm wide, thin- to somewhat thick-walled (walls up to 0.7 μm thick). Subhymenium thin; subhymenial hyphae richly branched, 2–3.7 μm wide, thin-walled. Cystidia thin-walled, colourless, smooth to heavily encrusted, of four types: 1) capitate scarce to common, 15–30 × 3.5–5 μm; 2) capitulate not frequent, 23–35 × 3–4.5 μm; 3) cylindrical and subcylindrical scattered, 20–28 × 3–4.3 μm; 4) fusoid rare, 25–35 × 4.5–5 μm. Incrustation on cystidia and basidioles partly dissolving in KOH. Basidioles short cylindrical to clavate, 10–13 × (3–)3.5–4.7 μm, smooth or slightly encrusted. Basidia cylindrical-utriform, 10.5–15.5 × 3.2–4 μm, smooth or slightly encrusted; sterigmata four, reaching 2.5–3 × 0.3–0.5 μm. Basidiospores ellipsoid to narrowly ellipsoid, 4–5(–5.5) × 2.7–3(–3.5) μm (L = 5.1 μm, W = 3.3 μm), thin-walled, smooth, colourless, some with a drop inside, Mz–, acyanophilous; apiculus minute, short.

##### Distribution.

So far, only known from Panama.

##### Ecology.

The species grows on small-sized dead corticated wood in tropical evergreen forests.

##### Notes.

The main diagnostic features of this species are a minutely warted hymenial surface, thickened walls in the subicular hyphae, the presence of smooth to heavily encrusted capitate and cylindrical cystidia, and thin-walled, narrowly ellipsoid basidiospores. This species is most morphologically similar to *L.sambuci*, *L.densiusculus*, and *L.denudatus*. It differs from *L.sambuci* s.str. by the presence of heavily encrusted capitulate cystidia and thin-walled basidiospores. The presence of richly encrusted capitulate cystidia and smaller basidiospores are the characteristics which separate *L.boquetensis* from *L.densiusculus*. Warted hymenial surface, cylindrical cystidia, and smaller basidiospores differentiate the new species from *L.denudatus*. The new species differs from a phylogenetically close *L.lancangjiangensis* by shorter and narrower basidiospores.

#### ﻿ Lyomyces granulosus

Taxon classificationFungiCorticialesHymenochaetales

Yurchenko & Langer sp. nov.

DE380791-3EC8-5F67-9881-300C00AC8445

850098

**Figure 5.**
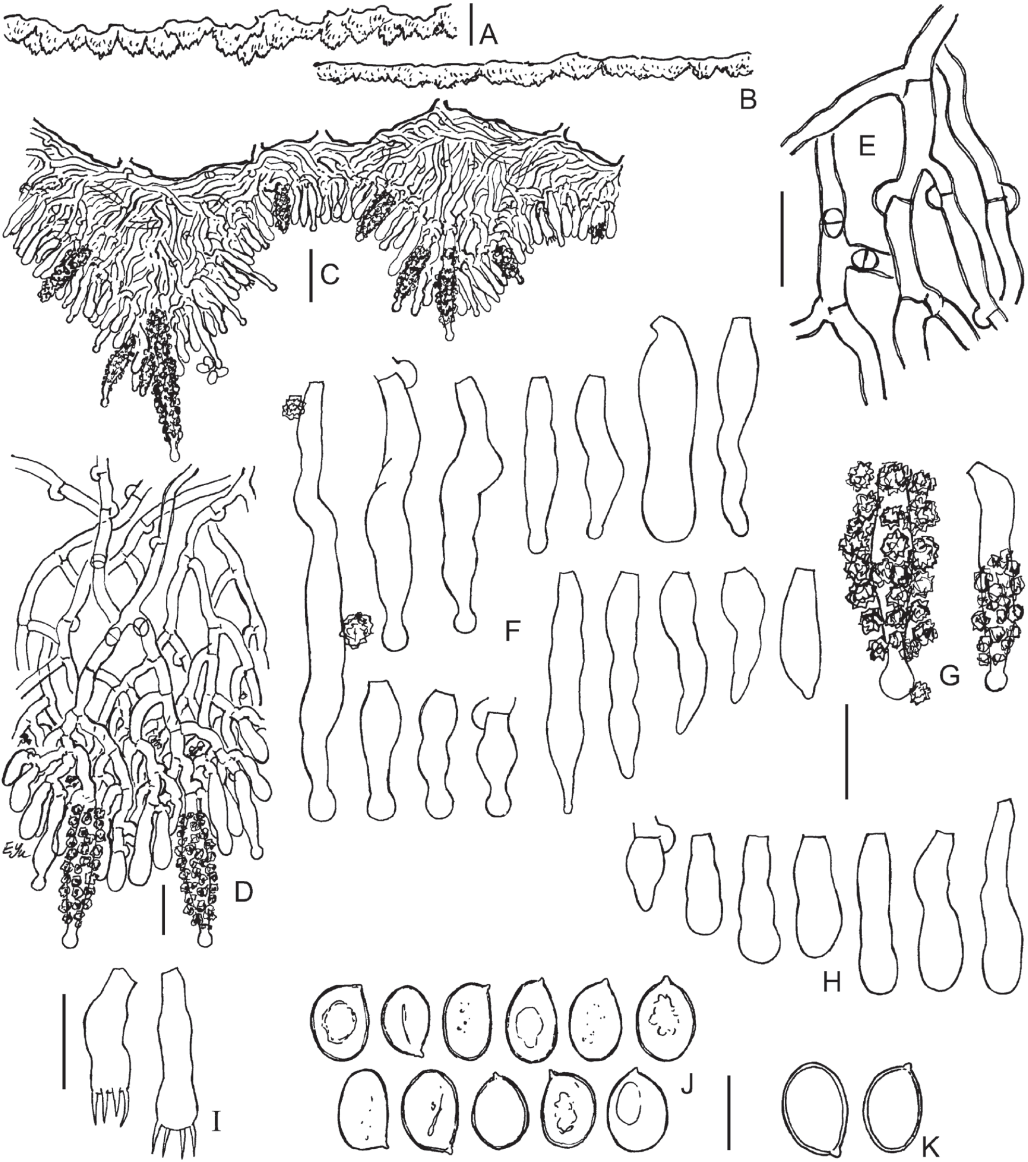
Micromorphology of *Lyomycesgranulosus* (holotype, KAS-GEL1662) **A–D** vertical sections through basidiomata **E** subicular hyphae **F** cystidia in KOH **G** cystidia in Mz**H** basidioles **I** basidia **J** basidiospores in KOH **K** basidiospores in Mz. Scale bars: 100 μm (**A, B**); 20 μm (**C**); 10 μm (**D–I**); 5 μm (**J, K**).

Figs 3C, D , 5 , 16C

##### Type.

Costa Rica • Alajuela Province: near Fraijanes and Poás volcano, 1940 m a.s.l., secondary evergreen tropical forest in a ravine, on wood and bark of dead branch or stem 30 mm in diam., 11 Feb 1989, E. Langer, G. Wagner No. 223 (***holotype***: KAS-GEL1662; ***isotype***: CFMR). GenBank: ITS = PP471799.

##### Etymology.

*granulosus* (Lat.) = having a grainy structure, due to the morphology of basidioma.

##### Description.

Basidiomata effused, 0.1–3 cm in extent, grandinioid-farinaceous to membranaceous, soft and fragile, usually consisting of as-if-confluent grains, 50–115 μm thick. Hymenial surface colliculose, whitish or creamish. Margin concolourous, pruinose, diffuse, 0.3–1 mm wide. Hyphal system monomitic, hyphae clamped at all septa, thin-walled, colourless. Subiculum thin, little differentiated from subhymenium. Subicular hyphae moderately branched, comparatively short-celled, 1.8–3.5(–4) μm wide, smooth; subhymenial hyphae richly branched, 2–3.2 μm wide, smooth or scarcely encrusted. Cystidia of several types: 1) capitate and subcapitate common, 15–47 × 4–5.5 μm, often with several slight constrictions, smooth to richly encrusted in the lower 2/3 of their length; 2) fusoid and subcylindrical scattered, 30–55 × 3–4.5(–6) μm, smooth to richly encrusted. Basidioles mostly clavate, 7.5–20 × 3.5–5(–6) μm, smooth. Basidia 14–19 × 4.5–4.7 μm, smooth, with four sterigmata 2–3 × 0.5–0.7 μm. Basidiospores broadly ellipsoid, (4.5–)5–5.5(–6.3) × (3.3–)3.5–4 μm (in holotype L = 5.1 μm, W = 3.7 μm), Q = 1.2–1.4(–1.6), thin- to often slightly thick-walled, smooth, colourless, Mz–, acyanophilous to slightly cyanophilous; apiculus small, but distinct.

##### Distribution.

So far, known from Costa Rica and Panama.

##### Ecology.

The species grows on dead decorticated small-sized wood in evergreen tropical forests, with a supposed preference for mountain ravines.

##### Additional specimens examined

**(*paratypes*).** Costa Rica • The same locality as holotype, on wood and bark of dead stem 7–15 mm in diam., 11 Feb 1989, E. Langer, G. Wagner (KAS-GEL1650); Panama • Chiriquí Province: W of Boquete town, Bajo Boquete community, 08°46.58'N, 082°28.17'W, 1450 m a.s.l., evergreen montane tropical forest, bottom of canyon with a rivulet, on a decorticated fallen branch, 27 Jul 2019, E. Yurchenko EYu 190727-8b (BLS M-2975; GenBank: ITS = PP471798, 28S = PP471819).

##### Notes.

The main diagnostic features of this species are the granulose structure of basidioma, thin-walled hyphae, presence of slightly constricted capitate cystidia, and broadly ellipsoid basidiospores with thin or slightly thickened walls. The species is morphologically close to *L.sambuci*, *L.denudatus*, and *L.fimbriatus*. In contrast to the new species, *L.sambuci* has less encrusted capitate cystidia and spores often with a drop inside. *Lyomycesfimbriatus* and *L.denudatus* have larger, thin-walled spores. Moreover, *L.fimbriatus* has an odontoid hymenial surface.

#### ﻿ Lyomyces napoensis

Taxon classificationFungiCorticialesHymenochaetales

Yurchenko & Riebesehl sp. nov.

4F65922D-75F7-575C-9F5A-482D03E03368

850099

**Figure 6.**
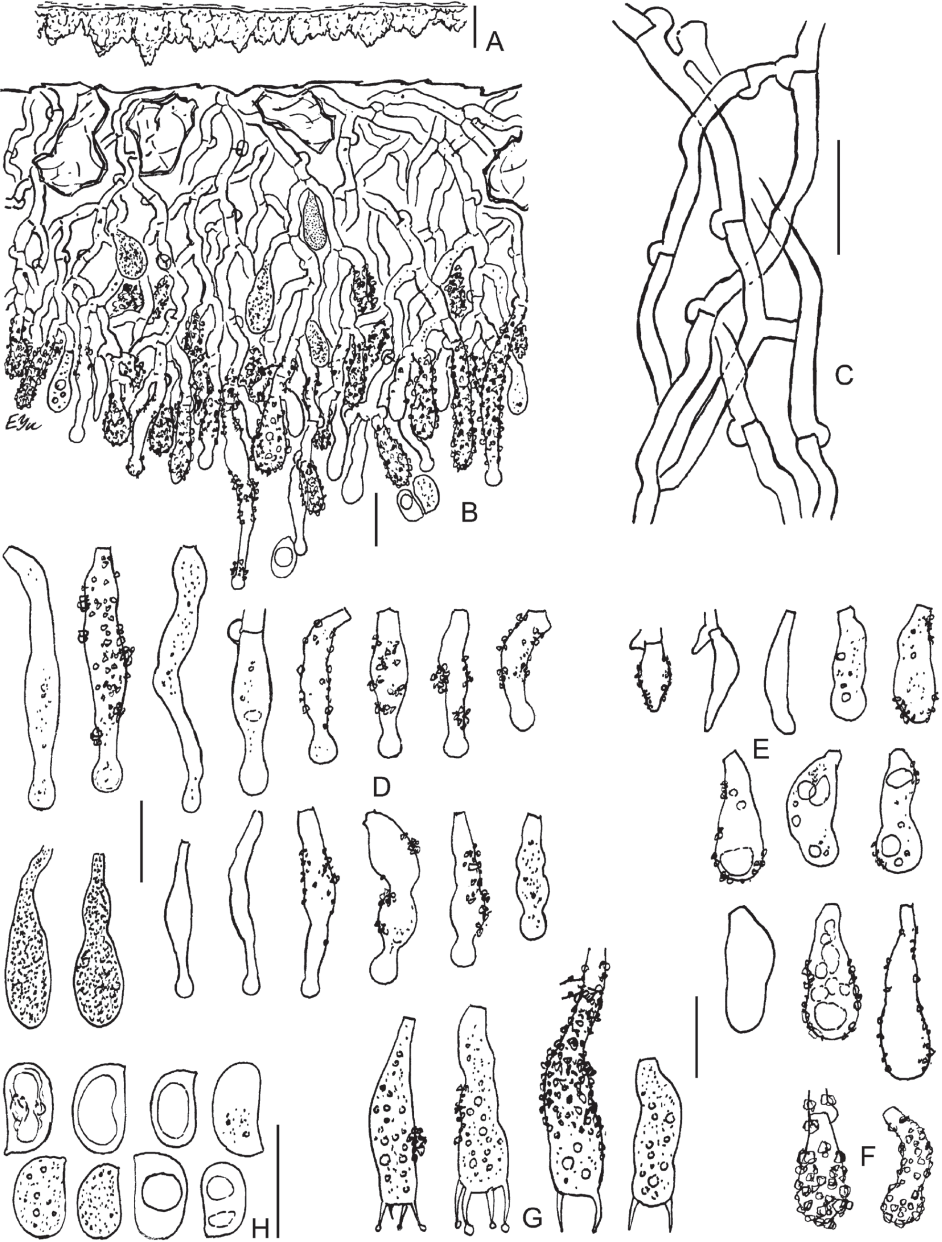
Micromorphology of *Lyomycesnapoensis* (holotype, BLS M-2610) **A, B** vertical sections through basidioma **C** subicular hyphae **D** cystidia **E** basidioles in KOH **F** basidioles in Mz**G** basidia **H** basidiospores. Scale bars: 100 μm (**A**); 10 μm (**B–H**).

Figs 3E, F , 6 , 16D

##### Type.

Ecuador • Orellana Province: between Puerto Francisco de Orellana (El Coca) and El Dorado, right bank of the Napo River, 00°29.17'S, 076°57.00'W, 260 m a.s.l., Amazonian rainforest, on dead standing lignified stem, 20 Jul 2019, E. Yurchenko EYu 190720-18 (***holotype***: BLS M-2610; ***isotype***: CFMR). GenBank: ITS = PP471800; 28S = PP471820.

##### Etymology.

The epithet refers to the Napo River, near which the type specimen was collected.

##### Description.

Basidiomata effused, 0.3–3.5 cm in extent, pruinose and mostly discontinuous to soft membranaceous, 45–90 μm thick. Hymenial surface dirty pale ochraceous, smooth to minutely warted; warts 3–5/mm, 30–45 μm high. Margin pruinose, diffuse (indeterminate), 0.3–2.5 mm wide. Hyphal system monomitic, hyphae moderately branched, clamped at all septa, thin-walled, colourless, smooth or encrusted. Cystidia and basidioles smooth to richly encrusted. Cystidia of several types: 1) capitate common, 15–35 × 3–5.5 μm, loosely encrusted except for capitate apex; 2) clavate cystidia with dark yellow granulose contents common, immersed, 12–23 × 5–7 μm; 3) fusoid and hyphoid cystidia rare or occasional, 18–26 × 4.3–5.5 μm. Basidioles short clavate to subcylindrical, 7.5–22 × 3–6(–7.3) μm, often with oily inclusions. Basidia utriform-cylindrical, 15.5–25 × 5.5–6.7 μm, slightly encrusted, with (2)4 sterigmata measuring 4–5 × 0.5–1 μm. Basidiospores narrowly ellipsoid to oblong, with adaxial side straight or slightly concave, 7–8.5(–9) × 4–4.7 μm (L = 7.1 μm, W = 4.4 μm), Q = 1.5–1.7, thin-walled, smooth, colourless, with oily inclusions, Mz–, acyanophilous; apiculus short and wide, or not pronounced.

##### Distribution.

So far, known from the northeast part of Ecuador, in the Amazonian Lowland.

##### Ecology.

The fungus grows on dead lignified stems, in equatorial rainforests.

##### Notes.

The species is distinguished from other members of *Lyomyces* by encrusted capitate cystidia, basidioles, and basidia, presence of clavate cystidia with dark yellow granulose contents, and by large, oblong, thin-walled basidiospores with oily contents. This species is morphologically close to *L.incrustatus* and *L.elaeidicola* because of the minute attached incrustation on basidioles and basidia. However, the two latter species have near-globose basidiospores.

#### ﻿ Lyomyces neocrustosus

Taxon classificationFungiCorticialesHymenochaetales

Yurchenko & Riebesehl sp. nov.

2861EBBB-BB45-5076-9E12-96BCB855C93B

850100

**Figure 7.**
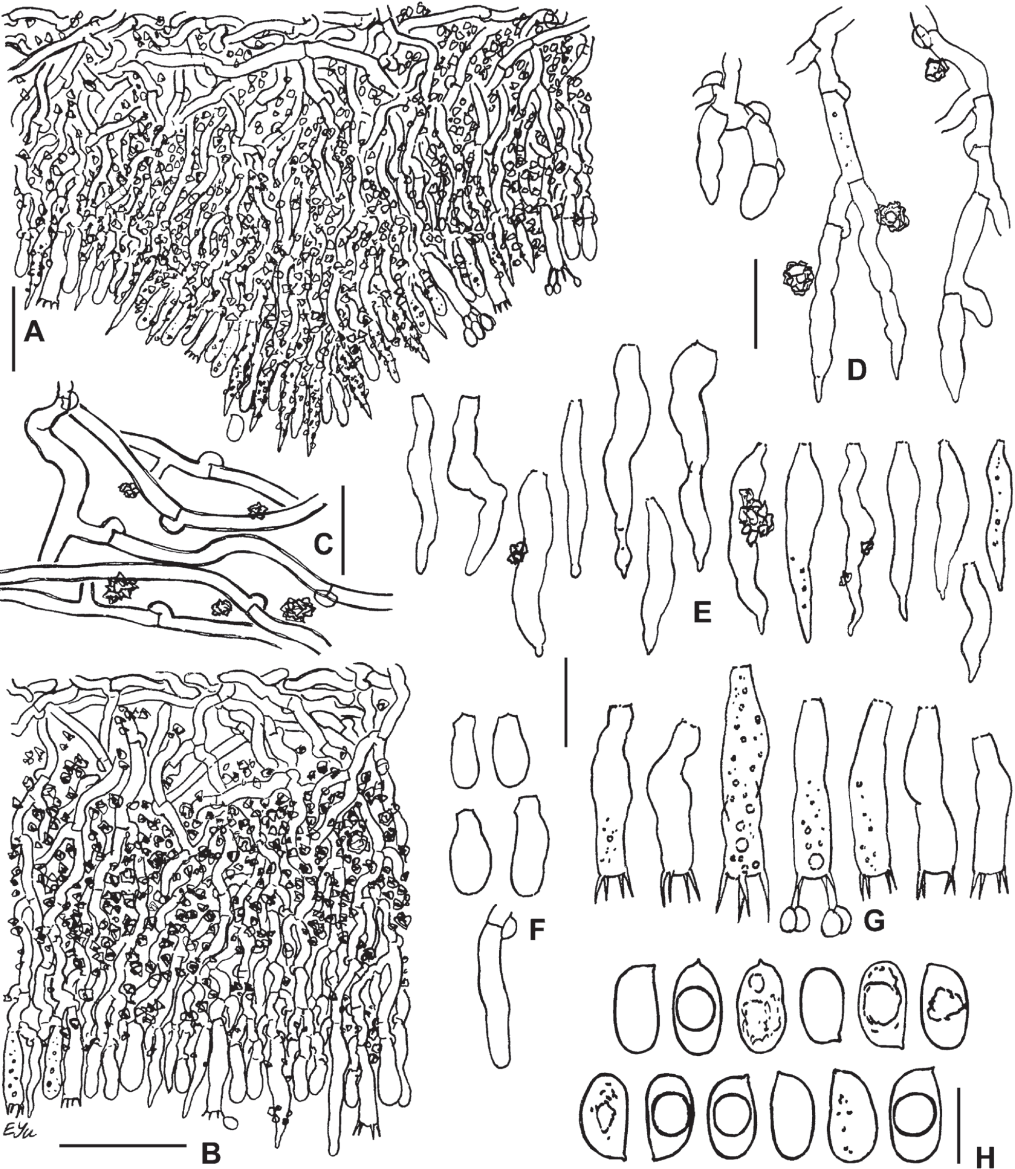
Micromorphology of *Lyomycesneocrustosus* (holotype, BLS M-5239) **A, B** vertical sections through basidioma **C** subicular hyphae **D** Subhymenial hyphae ended by cystidia and basidioles **E** cystidia **F** basidioles **G** basidia **H** basidiospores. Scale bars: 20 μm (A, **B**); 10 μm (**C–G**); 5 μm (**H**).

Figs 3G, H , 7 , 16E

##### Type.

Panama • Chiriquí Province: Bajo Mono NW of Boquete, southern slopes of Cordillera de Talamanca, near the Caldera River, 08°50.00'N, 082°28.83'W, about 1650 m a.s.l., edge or evergreen mountain rainforest, on small-sized dead trunk, mostly on the decorticated parts, 28 Jul 2019, E. Yurchenko EYu 190728-14 (***holotype***: BLS M-5239; ***isotype***: CFMR). GenBank: ITS = PP471801; 28S = PP471821.

##### Etymology.

*neo* + *crustosus* (Lat.) = (1) new in *L.crustosus* species complex, (2) a neotropical species, close to *L.crustosus*.

##### Description.

Basidiomata effused, 1.5–3 and more cm in extent, adnate, 25–110 μm thick excluding warts, membranaceous to subceraceous. Hymenial surface pale cream, white at the periphery, smooth to sparsely and minutely warted; warts up to 3–5/mm, 20–70 μm high. Margin paler than the main surface, from almost abrupt and pubescent to thinning out, 0.3–0.5(–1) mm wide. Hyphal system monomitic, hyphae clamped at all septa, thin-walled, colourless. Rosette-like crystals present in moderate or rich amounts between hyphae and hymenial elements. Subicular hyphae moderately branched, 2–3 μm wide; subhymenial hyphae richly branched, densely packed, 1.3–3.7(–4.5) μm wide. Subulate cystidia common, 16–25 × 2.5–3.5(–4.5) μm, seldom capitulate apically, smooth or slightly encrusted. Cylindrical cystidia rare, 18–21.5 × 2.7–3.5 μm. Basidioles narrowly ovoid to short clavate, seldom cylindrical, 11.5–18 × 3.8–4.5(–5.3) μm. Basidia subcylindrical to utriform, (13.5–)16–22.5 × (3.7–)4–4.5(–5.3) μm; sterigmata four, 2.5–5 × 0.7 μm. Basidiospores narrowly ellipsoid to oblong, (5.3–)5.5–6(–6.5) × (2.5–)3–3.5 μm (in holotype L = 5.75 μm, W = 3.2 μm), Q = (1.5–)1.7–1.8, thin- to slightly thick-walled (wall 0.3 μm thick), smooth, colourless, often with a large drop inside, Mz–, acyanophilous; apiculus short, but distinct.

##### Distribution.

Known from Panama and Colombia.

##### Ecology.

The species grows on dead wood in evergreen tropical forests.

##### Notes.

This species differs from *L.crustosus* by virtue of sparse and little-developed hymenophoral projections, the paler colour of the hymenial surface, thin-walled hyphae, shorter subulate cystidia, shorter basidioles, and broader, often slightly thick-walled basidiospores with a straight or convex adaxial side. In the two examined specimens, referred by us to *L.crustosus* s.str. (KAS-GEL2325, KAS-GEL5360; Fig. 1), the subulate cystidia were (20–)25–40(–50) μm long, basidioles were clavate to cylindrical, (13.5–)15–25 μm long, and basidiospores were 2.5–2.8(–3) μm wide, thin-walled, with straight or slightly concave adaxial side (in KAS-GEL5360 L = 5.6 μm, W = 2.7 μm). According to the illustrations in Eriksson and Ryvarden (1976), in *L.crustosus*, subulate cystidia are 22–40 μm long, and basidia are 23–33 μm long, which is about 1.5 times longer than in *L.neocrustosus*.

##### Additional specimens examined.

*Lyomycesneocrustosus*—Colombia • Magdalena Department: Tayrona National Natural Park, Estación de Gairaca, on dead corticated wood, 12 Jun 1978, L. Ryvarden 15737 (O-F-918425, ***paratype***).

*Lyomycescrustosus*—Germany • Bayern: Oberallgäu district, Hinterstein vicinity, about 850 m a.s.l., on a dead twig of cf. *Fraxinusexcelsior*, 19 Sep 1991, G. Langer, E. Langer (KAS-GEL2325) • Hinterstein vicinity, about 1060 m a.s.l., on dead wood of *Fagussylvatica*, 14 Oct 1998, E. Langer, E. Hennen (KAS-GEL5360).

#### ﻿ Lyomyces oleifer

Taxon classificationFungiCorticialesHymenochaetales

Yurchenko & Langer sp. nov.

3A712730-F00C-5F24-B5E2-3B062BDEBA09

850101

Figs 8A , 9

##### Type.

Ecuador • Zamora Chinchipe Province: 17 km NW of Zamora, in the vicinity of Estación Científica San Francisco, Cordillera Consuelo Mountain Range, Permanent sample plot No. 3, 03°58.70'S, 079°04.43'W, about 2150 m a.s.l., on corticated parts of a dead still-attached twig 8–10 mm in diam., 28 Aug 2001, E. Langer 7/47 (***holotype***: KAS-Ec47-2001; ***isotype***: CFMR). GenBank: ITS = PP471802.

##### Etymology.

*oleifer* (Lat.) = bearing oil, due to numerous drops of oily substance visible in microscopic sections of basidiomata.

##### Description.

Basidiomata effused, adnate, membranaceous, 0.2–5 and more cm in extent, between aculei from minutely porulose to continuous, 60–125 μm thick, cracking with age. Hymenial surface odontoid, cream-coloured or yellowish; aculei conical to subcylindrical, 5–7/mm, 45–115 μm high, 20–65 μm in diam. Margin more or less concolourous, abrupt, narrowly mould-like (finely fimbriate) or thinning out, 0.2–0.5 mm wide. Hyphal system monomitic, hyphae clamped at all septa, colourless. Subicular hyphae 1.7–3.3 μm wide, thin- to somewhat thick-walled. Subhymenial hyphae densely agglutinated, 1.5–3.2 μm wide, thin-walled. Hymenial elements mostly indistinct due to the presence of oily matter in the hymenium. Cystidia smooth or encrusted, of several types: 1) capitulate or subcapitate common, 20–40(–45) × 2.5–4 μm, with apex 1.5–1.7 μm wide; 2) fusoid common, 25–30(–48) × 2.3–4.7 μm, apically blunt, occasionally acute; 3) cylindrical rare, 20–30 × 2.3–4.3 μm. Oily substance in the shape of drops, droplets, or amorphous masses present in hymenium or near cystidia in preparations (KOH). Basidioles clavate to subcylindrical, 13–30 × 3.5–5.5 μm. Basidia long-utriform, 20–25 × 4–4.5 μm; sterigmata four, 3.5–4 × 0.7–1 μm. Basidiospores narrowly ellipsoid to oblong, 5.5–6.5(–7) × 3.2–4 μm (in holotype L = 5.8 μm, W = 3.5 μm), Q = 1.6–1.7, slightly thick-walled, smooth, colourless, often with a drop inside, Mz–, acyanophilous; apiculus middle-sized.

**Figure 8.**
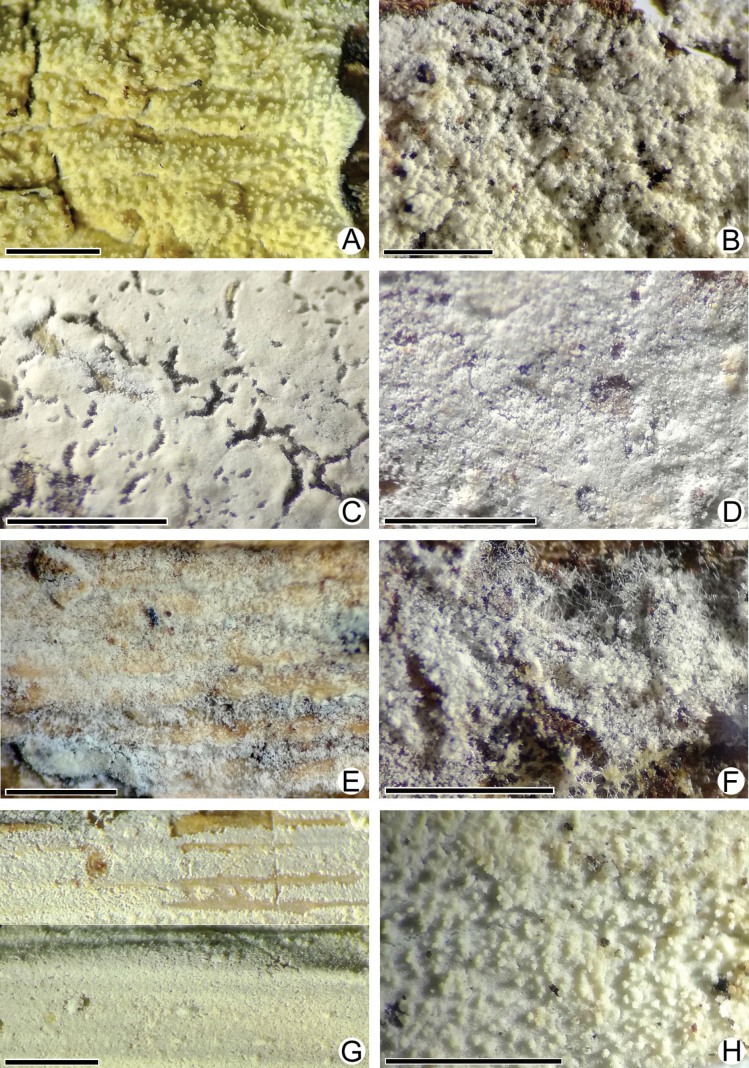
Macromorphology of *Lyomyces* species **A***L.oleifer*, holotype (KAS-Ec47-2001) **B***L.orarius*, holotype (BLS M-2995) **C***L.pantropicus*, holotype (BLS M-2679) **D***L.pantropicus*, BLS M-3545 **E***L.parvus*, holotype (KAS-GEL1599) **F***L.parvus*, BLS M-2990 **G***L.sceptrifer*, holotype (KAS-Ec661-2002) **H***L.subcylindricus*, holotype (BLS M-2668). Scale bars: 1 mm.

**Figure 9.**
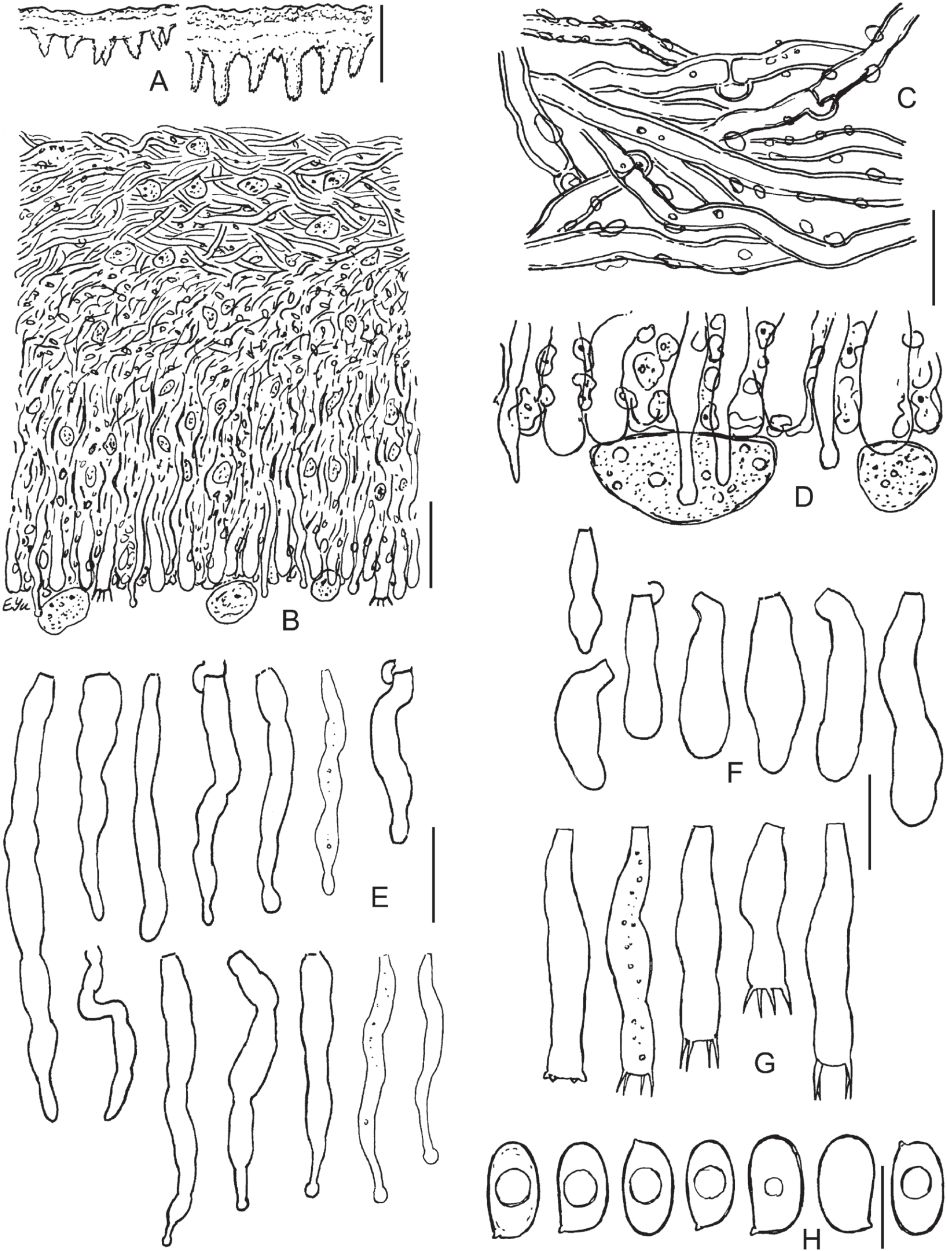
Micromorphology of *Lyomycesoleifer* (holotype, KAS-Ec47-2001) **A, B** vertical sections through basidioma **C** subicular hyphae **D** upper part of hymenium with oildrops **E** cystidia **F** basidioles **G** basidia **H** basidiospores. Scale bars: 100 μm (**A**); 20 μm (**B**); 10 μm (**C–G**); 5 μm (**H**).

##### Distribution.

Known from the Andes Mountains in southern Ecuador.

##### Ecology.

The species grows on dead twigs in evergreen tropical montane forests.

##### Additional specimens examined

**(*paratypes*).** Ecuador • Zamora Chinchipe Province: 17 km NW of Zamora, in the vicinity of Estación Científica San Francisco, between 1850 and 2450 m a.s.l., on dead corticated twigs 3–7 mm in diam., 3 Sep 2001, E. Langer 7/287 (KAS-Ec287-2001; GenBank: ITS = PP471803); • ibid., on a dead corticated branch, 8 Sep 2001, E. Langer 1/423 (KAS-Ec423-2001; duplicate: CFMR; GenBank: ITS = PP471804).

##### Notes.

This new species differs from other species of *Lyomyces* by drops of oily substance, released in slides from all layers of basidioma. The size, shape, and refractive properties of the oily matter from the hymenium in this fungus depend on the age of basidioma. The new species differs from the most morphologically close *L.crustosus* by densely odontoid basidioma, numerous capitulate cystidia, tapering cystidia with mostly blunt apices, long-utriform basidia, and slightly thick-walled basidiospores. Besides the main types of cystidia described above, the intermediate forms between them are present in the specimens. In a paratype (KAS-Ec423-2001), the spores have L = 5.9 μm and W = 3.5 μm. For KAS-Ec287-2001 and KAS-Ec423-2001, the features of white rot of the wood, associated with the basidiomata, were noted in the field.

#### ﻿ Lyomyces orarius

Taxon classificationFungiCorticialesHymenochaetales

Yurchenko & Riebesehl sp. nov.

9032940D-42A5-5923-832C-7DE477F7FEEA

850102

Figs 8B , 10

##### Type.

Ecuador • Esmeraldas Province: NW of Esmeraldas town, Pacific Ocean shore, 00°59.13'N, 079°40.15'W, 5–10 m a.s.l., semi-xerophytic bush vegetation on a coastal hill, on the bark of a dead stump, 24 Jul 2019, E. Yurchenko EYu 190724-1 (***holotype***: BLS M-2995; ***isotype***: CFMR). GenBank: ITS = PP471805; 28S = PP471822.

##### Etymology.

*orarius* (Lat.) = occurring on seashore, referring to the geography of type locality.

##### Description.

Basidiomata effused, 3–20 mm in extent, 70–100 μm thick, soft-membranaceous, porulose to minutely grandinioid; hymenial surface white or greyish; margin almost abrupt to diffuse, and then up to 0.3 mm wide. Hyphal system monomitic, hyphae clamped at all septa, moderately branched, colourless. Subiculum loose; subicular hyphae 1.8–3.2 µm wide, often branched at a right angle, with thin- or slightly-thickened walls, naked or slightly encrusted. Subhymenial texture mostly masked by crystalline material; subhymenial hyphae 2–4 µm wide, thin-walled. Cystidia of several types: 1) capitate, subcapitate, capitulate, often projecting, 15–37 × 3.3–4 μm, moderately to richly encrusted in the lower 2/3s of their length, rarely naked, capitate/capitulate ends 2–3 μm wide, occasionally with a cap of resinous matter 4–6 μm wide, the latter easily detached in preparation; 2) cylindrical, 15–33 × 3–3.5 μm; 3) tapering, hypha-like, supposedly the collapsed stages of capitate and cylindrical variants, 20–33 × 2.7–3.2 μm. Basidioles irregularly cylindrical to subclavate, 8.5–12.5 × 3–3.8 μm, smooth to richly encrusted. Basidiospores ellipsoid to narrowly ellipsoid, 4.3–5(–5.5) × (2.5–)2.8–3.2(–3.5) µm (L = 4.55 µm, W = 3.1 µm), Q = 1.5–1.7, with thin- or unclearly thickened wall, smooth, colourless, sometimes with a drop inside; apiculus minute or indistinct.

**Figure 10.**
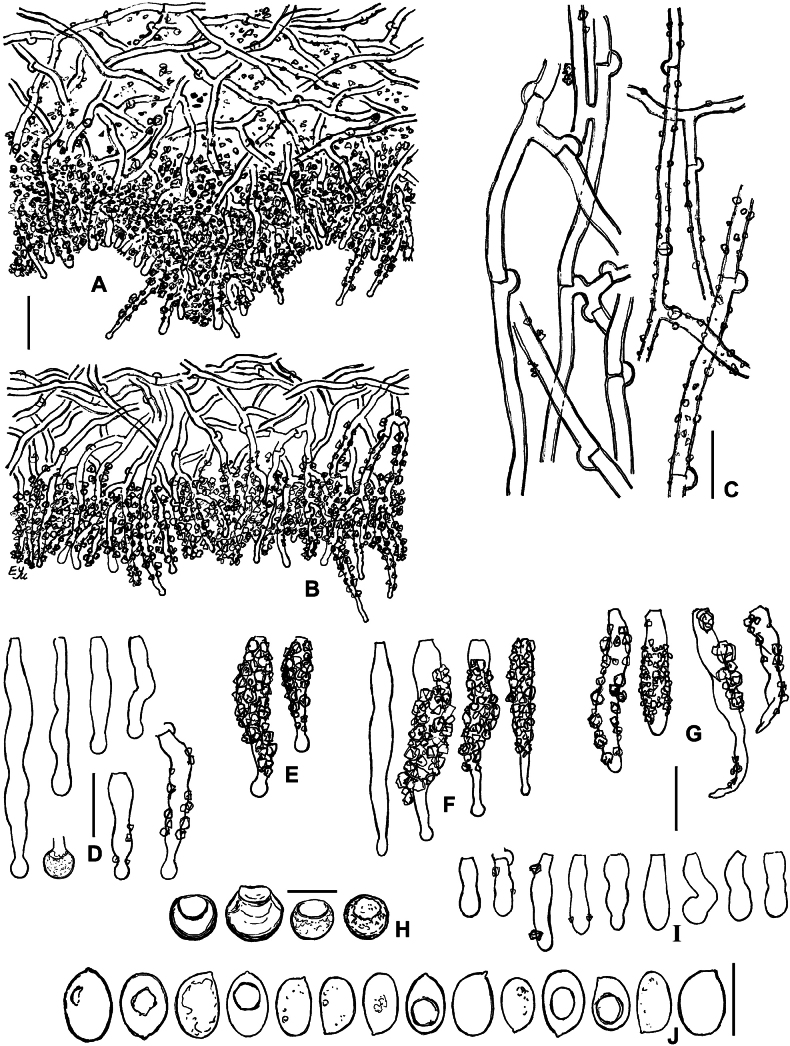
Micromorphology of *Lyomycesorarius* (holotype, BLS M-2995) **A, B** vertical sections through basidioma **C** subicular hyphae **D** capitate cystidia in KOH **E** capitate cystidia in Mz**F** capitulate cystidia **G** cylindrical and tapering cystidia **H** caps of resinous matter detached from capitate cystidia **I** basidioles **J** basidiospores. Scale bars: 20 μm (**A, B**); 10 μm (**C–G, I**); 5 μm (**H, J**).

##### Distribution.

So far, known from the northwest of Ecuador.

##### Ecology.

The species grows on dead corticated wood in tropical monsoon semi-deciduous forests and bushes.

##### Notes.

Mature basidia with sterigmata were not found in the holotype. The species is most morphologically close to *L.sambuci* and *L.bambusinus*. The new species is distinguished from *L.sambuci* by smaller, mostly thin-walled basidiospores and the presence of capitulate cystidia, richly encrusted in the lower 2/3s of their length. In contrast to *L.orarius*, *L.bambusinus* has thick-walled hyphae, little-encrusted capitate cystidia, long (40–65 μm) tapering cystidia, subulate cystidioles (12–17 μm long), and larger (up to 6.2 × 4.8 μm) spores with constantly thickened walls (Chen and Zhao 2020).

#### ﻿ Lyomyces pantropicus

Taxon classificationFungiCorticialesHymenochaetales

Yurchenko & Riebesehl sp. nov.

BD853F80-71EA-5010-A674-7372F2F711F9

850105

**Figure 11.**
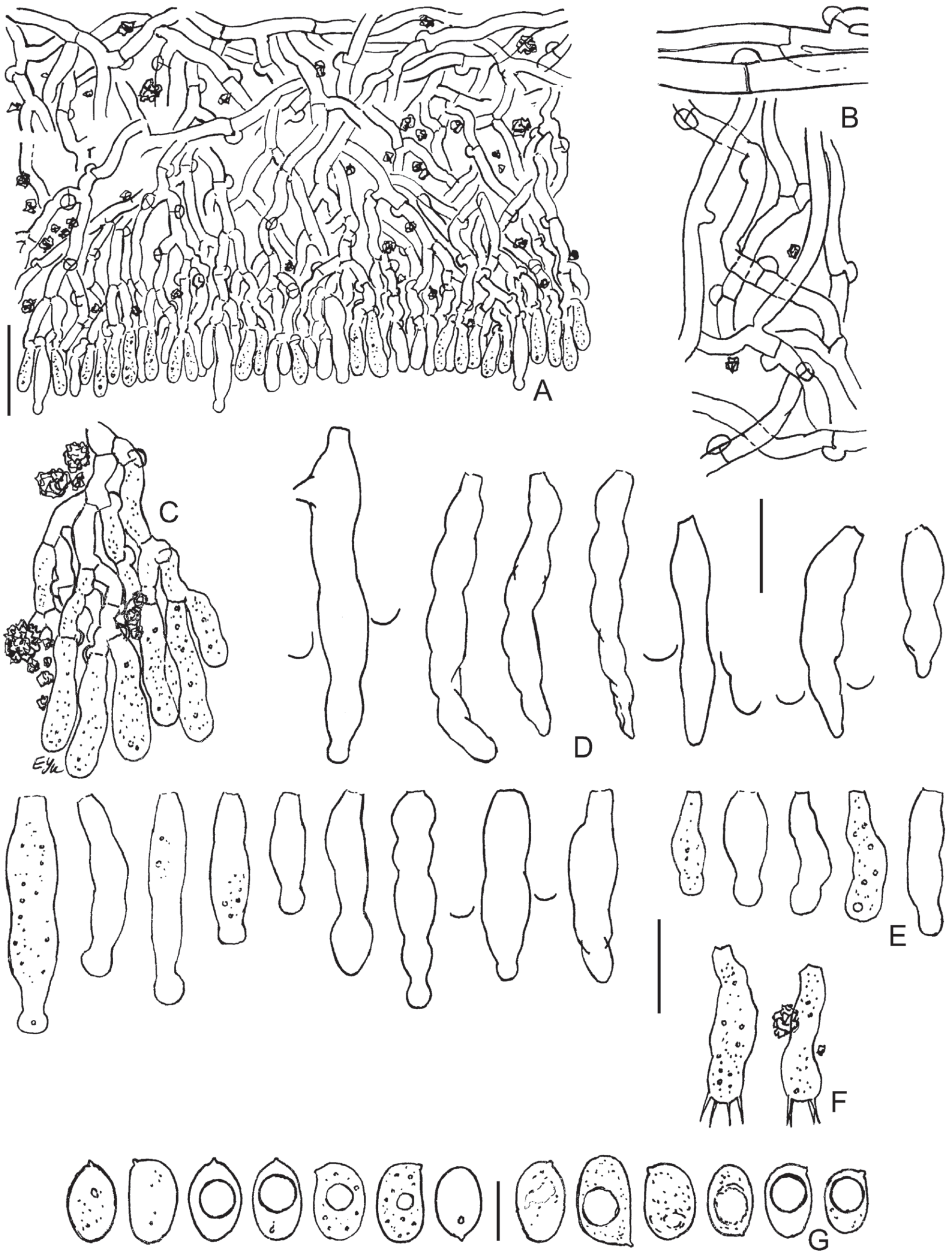
Micromorphology of *Lyomycespantropicus* (holotype, BLS M-2679) **A** vertical section through basidioma **B** subicular hyphae **C** portion of hymenium and subhymenium **D** cystidia **E** basidioles **F** basidia **G** basidiospores. Scale bars: 20 μm (**A**); 10 μm (**B–G**).

**Figure 12.**
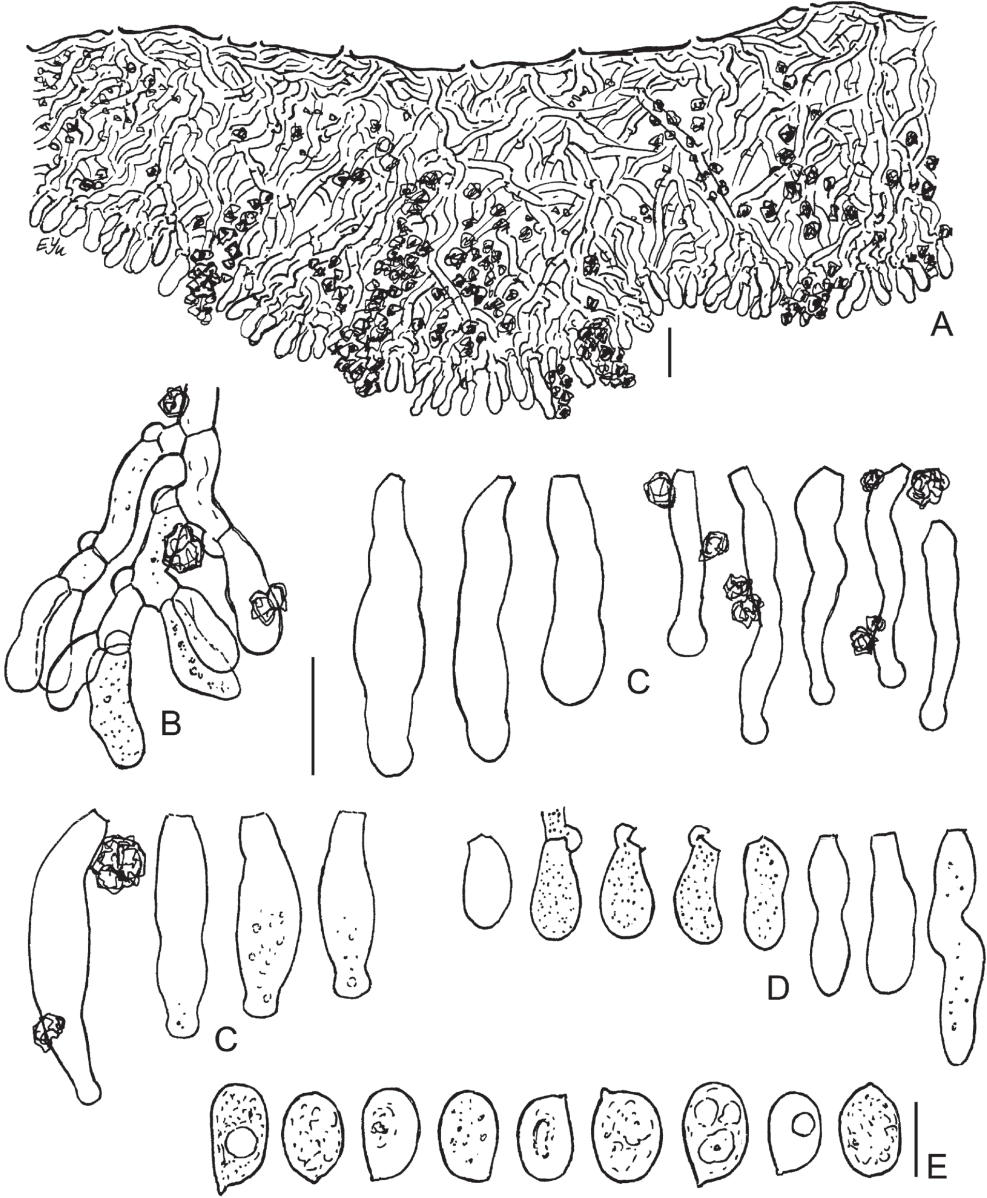
Micromorphology of *Lyomycespantropicus* (BLS M-3447) **A** vertical section through basidioma **B** portion of hymenium and subhymenium **C** cystidia **D** basidioles **E** basidiospores. Scale bars: 10 μm.

Figs 8C, D , 11 , 12 , 16F

##### Type.

Panama • Chiriquí Province: W of Boquete town, Bajo Boquete community, 08°46.45'N, 082°28.03'W, about 1400 m a.s.l., evergreen montane tropical mixed forest (with *Pinus*), on fallen decorticated bush stem, 27 Jul 2019, E. Yurchenko EYu 190727-23b (***holotype***: BLS M-2679; ***isotype***: CFMR). GenBank: ITS = PP471808; 28S = PP471825.

##### Etymology.

The epithet refers to the extended character of species’ distribution in the tropics: *pan*- (Lat.) = throughout.

##### Description.

Basidiomata effused, 0.1–5 and more cm in extent, adnate, membranaceous, cracking with age, continuous to discontinuous-porulose to the periphery, 35–85 μm thick. Hymenial surface from furfuraceous to usually smooth, rarely colliculose, white or greyish. Margin concolourous, thinning out or diffuse, 0.5–1.5 mm wide. Hyphal system monomitic, hyphae clamped at all septa, thin-walled, colourless, smooth or loosely encrusted by crystals or their aggregates 2–5(–10) μm in size. Subicular hyphae moderately branched, disordered or subhorizontal, (1.7–)2.3–3.2 μm wide. Subhymenium little differentiated from subiculum; subhymenial hyphae moderately to richly branched, with cylindrical or sometimes short swollen segments, 2–4(–5) μm wide. Hymenial elements smooth to moderately encrusted. Cystidia inconspicuous, thin-walled, immersed or slightly protruding over other hymenial elements, of four types: 1) irregularly cylindrical scattered, 20–35 × 4–5.5 μm; 2) subcapitate and capitate scattered, 13–23 × 3.5–5 μm, with the capitate end often slightly flattened from the top; 3) fusoid scarce, 16–23 × 5 μm; 4) hyphidia-like occasional, 13–27 × 2.5–3 μm. Basidioles subcylindrical, narrowly clavate, rarely clavate, subcapitate, utriform, (9–)11–25.5 × 3.3–4.5(–5) μm, often minutely guttulate or granulose inside. Basidia subcylindrical, subclavate, utriform, 16–17(–22) × 4–4.5(–5) μm; sterigmata four, 3–4.5 × 0.5–0.7 μm. Basidiospores ellipsoid to oblong, (5–)5.5–7(–7.5) × (3.2–)3.5–4.5 μm (in holotype L = 6.5 μm, W = 4.3 μm), Q = (1.3–)1.4–1.7, with thin or barely thickened wall, smooth, colourless, with granulose or heterogeneous contents and often with a large oildrop, Mz–, acyanophilous; apiculus minute or indistinct.

##### Distribution.

Our collections originate from the western part of Panama and from the northwest, northeast, and southern parts of Ecuador. Supposedly, it is a common species in Central and South America. The ITS alignment and phylogenetic inference results suggest that the distribution range of this new species also includes Southeast Asia (China and Sri Lanka).

##### Ecology.

The fungus grows on dead branches and various wooden stems of angiosperms in humid and semi-arid, angiosperm and mixed tropical forests, from sea level to about 1900 m a.s.l.

##### Additional specimens examined

**(*paratypes*).** Panama • Chiriquí Province: W of Boquete town, Bajo Boquete community, 08°46.53'N, 082°27.87'W, 1450 m a.s.l., mixed (with *Pinus*) evergreen montane tropical forest, on a dead corticated woody stem, 27 Jul 2019, E. Yurchenko EYu 190727-5c (BLS M-9943); Ecuador • Zamora Chinchipe Province: 17 km NW of Zamora, in the vicinity of Estación Científica San Francisco, Permanent sample plot No. 5, 03°58.38'S, 079°04.65'W, 1860 m a.s.l., on dead bamboo stem, 8 Mar 2002, E. Langer 1/727 (KAS-Ec727-2002); • Orellana Province: near Puerto Francisco de Orellana (El Coca), S of Flor de Oriente village, 00°30.25'S, 076°58.92'W, 295 m a.s.l., equatorial rainforest on hilly relief, on a fallen decorticated branch, 21 Jul 2019, E. Yurchenko EYu 190721-12 (BLS M-3484); • Esmeraldas Province: NW of Esmeraldas town, Pacific Ocean shore, 00°59.13'N, 079°40.15'W, 5–10 m a.s.l., semi-xerophytic bush vegetation on a coastal hill, on a dead corticated still-attached branch, 24 Jul 2019, E. Yurchenko EYu 190724-12 (BLS M-3447; GenBank: ITS = PP471807; 28S = PP471824); • ibid., on a mostly corticated fallen twig, 24 Jul 2019, E. Yurchenko EYu 190724-16 (BLS M-3406); • ibid., on fallen corticated branches, 24 Jul 2019, E. Yurchenko EYu 190724-17 (BLS M-3545).

##### Notes.

The main diagnostic features of this species are membranaceous basidiomata with slightly furfuraceous to usually smooth hymenial surface, thin-walled hyphae, presence of inconspicuous, scattered, irregularly cylindrical and subcapitate cystidia, large crystalline aggregates in basidioma, and relatively large basidiospores with a thin or barely thickened wall. This species is phylogenetically very close to *L.microfasciculatus*, but in contrast to the latter, the hymenial surface of *L.pantropicus* lacks any projections like warts or aculei. Further *L.pantropicus* has morphologically much more diverse cystidia, and larger spores. In morphology, *L.pantropicus* is most similar to *L.gatesiae* due to the presence of slightly constricted, subcylindrical cystidia, large crystals on the hyphae, and spores with one large drop inside. From the latter species, it differs by ellipsoid spores and the lack of pigmentation of the hymenial surface. The new species is differentiated from *L.sambuci* and *L.densiusculus* by slightly apically flattened capitate ends of cystidia.

This fungus can develop fairly large basidiomata and their groups, e.g., in KAS-Ec727-2002 there is a notation that fruiting bodies cover 90 cm of stem length and provoke the features of white rot. Besides, KAS-Ec727-2002 can be noted as a variation of *L.pantropicus*, which almost entirely lacks crystalline deposits and has spindle-shaped cystidia.

#### ﻿ Lyomyces parvus

Taxon classificationFungiCorticialesHymenochaetales

Yurchenko & Langer sp. nov.

5D995F5C-E055-5660-BA6E-746A616E8865

850103

**Figure 13.**
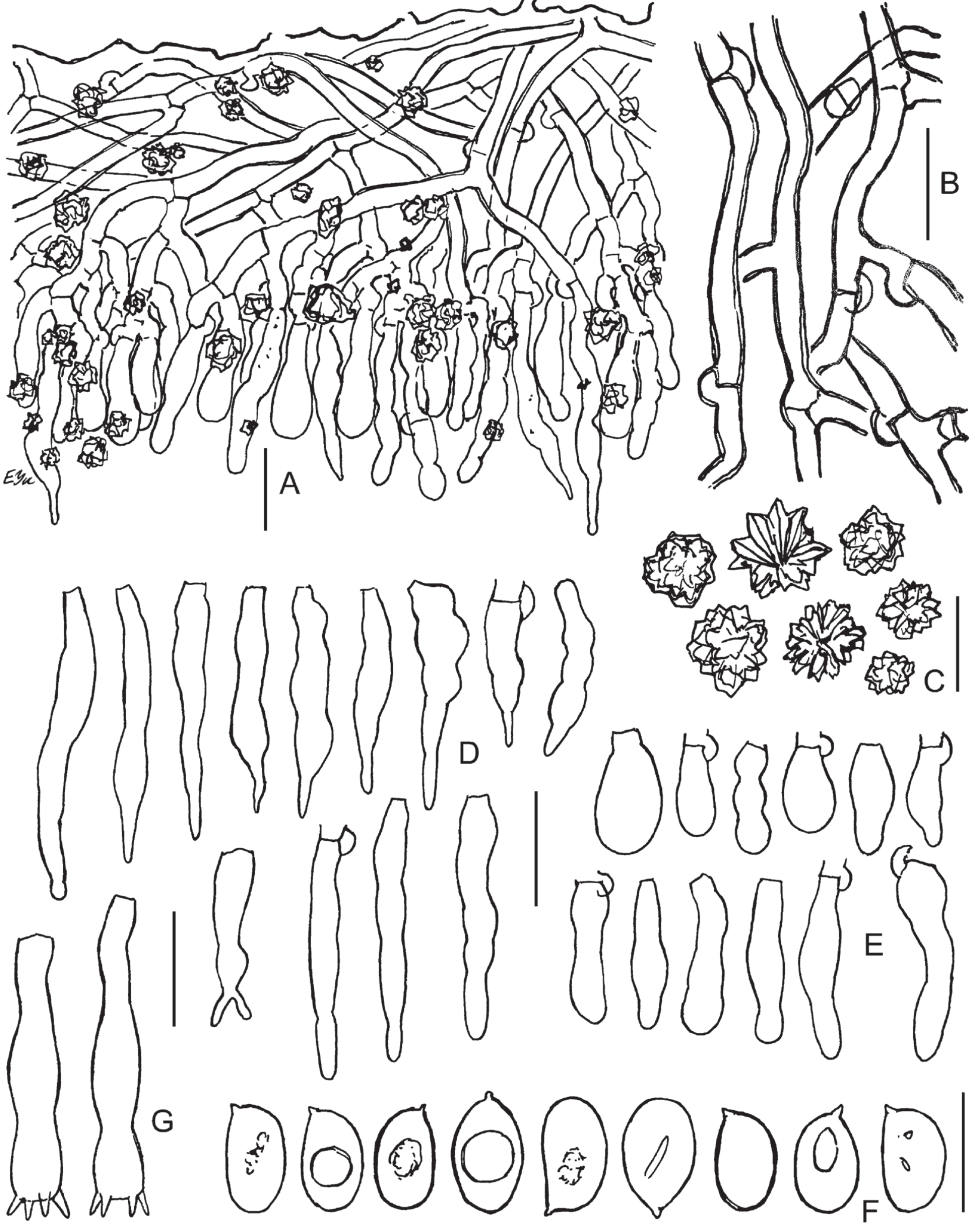
Micromorphology of *Lyomycesparvus* (holotype, KAS-GEL1599) **A** vertical section through basidioma **B** subicular hyphae **C** crystals from subiculum and subhymenium **D** cystidia **E** basidioles **F** basidiospores. BLS M-2990: **G** basidia. Scale bars: 10 μm (**A, B, D, E, G**); 5 μm (**C, F**).

Figs 8E, F , 13 , 16G

##### Type.

Costa Rica • Cartago Province: Orosí, Río Macho, secondary evergreen tropical forest near the Orosí River, 1140 m a.s.l., on dead naked wood, 10 Feb 1989, E. Langer, G. Wagner 97 (***holotype***: KAS-GEL1599; ***isotype***: CFMR). GenBank: ITS = PP471810.

##### Etymology.

*parvus* (Lat.) = minute, scanty, due to very thin and small-sized basidiomata.

##### Description.

Basidiomata effused, 0.1–2 and more cm in extent, 40–70 μm thick, mostly discontinuous, pruinose or farinaceous, in older areas submembranaceous, whitish, subinvisible when dry. Hymenial surface white, without projections. Margin diffuse, indeterminate, up to 0.5 mm wide. Hyphal system monomitic, hyphae clamped at all septa, thin-walled, colourless. Subicular hyphae loosely arranged, 1.5–2.8 μm wide. Subhymenium little differentiated from subiculum; subhymenial hyphae 1.8–3 μm wide. Scattered rosette-like, 2–5 μm in diam., loosely attached crystals present in subiculum, subhymenium, and hymenium. Cystidia smooth or encrusted by crystals, of several types: 1) fusoid to subulate and almost capitulate common, often with slight constrictions, sometimes branched above, 13–31.5 × 2.5–4.5 μm; 2) cylindrical rare, 15–23 × (2.2–)2.7–3.3 μm. Basidioles ovoid to short fusoid and subcylindrical, 7.5–17 × 3–5.5 μm. Basidia long-utriform, 23–27 × 3.8–4.5 μm; sterigmata four, 2.5–3 × 1.5 μm. Basidiospores ellipsoid to oblong, (3.7–)4–5(–5.3) × (2.3–)2.5–3.5 μm (in holotype L = 4.6 μm, W = 2.7 μm), Q = (1.4–)1.6–1.7(–1.8), smooth, thin-walled, colourless, some with a drop inside, Mz–, acyanophilous; apiculus small, short.

##### Distribution.

The species is so far known from Costa Rica and Ecuador.

##### Ecology.

The species grows on dead wood in evergreen angiosperm and mixed tropical forests at altitudes of about 1000–2700 m a.s.l.

##### Additional specimen examined

**(*paratype*).** Ecuador • Pichincha Province: Quito City, left slope of the Machángara River canyon, Guápulo Park, 00°11.77'S, 078°28.37'W, 2660 m a.s.l., small semi-natural forest with *Pinus*, on a fallen, mostly decorticated branch of *Pinus* sp., 18 Jul 2019, E. Yurchenko EYu 190718-23 (BLS M-2990). GenBank: ITS = PP471809; 28S = PP471826.

##### Notes.

The main diagnostic features of this species are very thin, loose basidiomata, hardly visible to the naked eye in a dry state, narrow thin-walled hyphae, presence of subulate and subcapitate cystidia, and ellipsoid, thin-walled basidiospores. The holotype of this species has oblong basidiospores (2.3–)2.5–3 μm wide, whereas spores in the paratype are ellipsoid, (2.5–)2.8–3.5 μm wide. Other morphological features of both specimens are identical. This species is close to *L.crustosus* and *L.juniperi* in the micromorphology of hyphae, hymenial elements, and spores, but has pruinose, discontinuous basidiomata.

#### ﻿ Lyomyces sceptrifer

Taxon classificationFungiCorticialesHymenochaetales

Yurchenko & Langer sp. nov.

06D79587-604A-5054-9E85-1203AD4A69E3

850104

Figs 8G , 14

##### Type.

Ecuador • Zamora Chinchipe Province: 17 km NW of Zamora, in the vicinity of Estación Científica San Francisco, near the San Francisco River, Permanent sample plot No. 1, 03°58.33'S, 079°04.67'W, about 1850 m a.s.l., on a dead bamboo stem, 5 Mar 2002, E. Langer 1/661 (***holotype***: KAS-Ec661-2002; ***isotype***: CFMR). GenBank: ITS = PP471811; 28S = PP471827.

##### Etymology.

*sceptrifer* (Lat.) = bearing sceptrum, referring to the shape of cystidia with a prominent capitulum and widened lower half.

##### Description.

Basidiomata effused, 5 and more cm in extent, 40–70 μm thick, loose- or soft- membranaceous. Hymenial surface cream-coloured, even, but under a lens very minutely porulose. Margin concolourous, from abrupt to thinning out and diffuse, up to 0.5 mm wide. Hyphal system monomitic, hyphae clamped at all septa, colourless, smooth. Subicular hyphae moderately branched, (1.5–)2–3.3 μm wide, thin- to slightly thick-walled. Subhymenial hyphae richly branched, 1.5–3.5 μm wide, thin-walled. Cystidia of three main types: 1) capitate numerous, 22–33 × 3.5–6, with long narrow neck and widened base, apically naked, seldom bearing a cap of transparent matter, hardly preserved in preparations; 2) cylindrical and irregularly-cylindrical scarce, (20–)30–65 × 3–7 μm; 3) clavate scarce, 30–35 × 8 μm. Basidioles clavate to utriform, (7.5–)9–16 × 3.5–4.7 μm. Basidia utriform, 16–18.5 × 4–5 μm, sterigmata four, 4–5.5 × 0.7–1 μm. Basidiospores ellipsoid, 4.3–4.8(–5) × 2.7–3(–3.5) μm (L = 4.7 μm, W = 2.9 μm), Q = 1.4–1.7, smooth, colourless, with thin or faintly thickened wall, Mz–, acyanophilous; apiculus well-pronounced.

**Figure 14.**
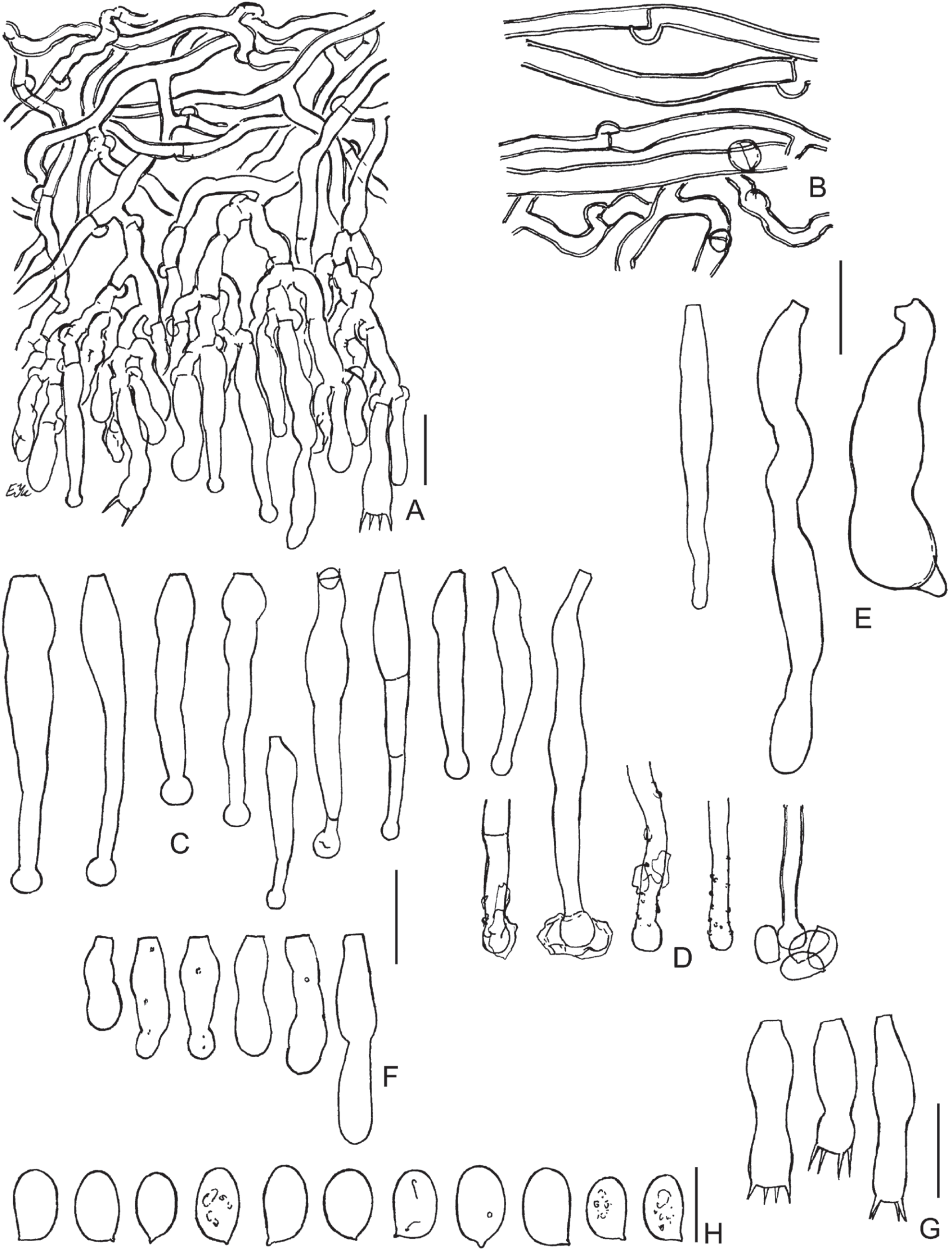
Micromorphology of *Lyomycessceptrifer* (holotype, KAS-Ec661-2002) **A** vertical section through basidioma **B** subicular hyphae **C** capitate cystidia **D** Incrustations and attached basidiospores on cystidia **E** Irregularly cylindrical cystidia **F** basidioles **G** basidia **H** basidiospores. Scale bars: 10 μm (**A–G**); 5 μm (**H**).

##### Distribution.

So far, known from the Andes Mountains in southern Ecuador.

##### Ecology.

The species grows on dead lignified stems in evergreen tropical montane forests.

##### Notes.

The main diagnostic features of this species are an even hymenial surface, smooth, slightly thick-walled subicular hyphae, smooth, capitate cystidia with long narrow neck, and ellipsoid basidiospores with thin- to faintly thickened walls and well-pronounced apiculus. The species differs from close taxon *L.sambuci* by the absence of crystalline incrustations on capitate cystidia and the presence of scattered, fairly large subcylindrical cystidia.

#### ﻿ Lyomyces subcylindricus

Taxon classificationFungiCorticialesHymenochaetales

Yurchenko & Riebesehl sp. nov.

A969D726-0D12-57BD-88C1-A3F0B498A945

850106

Figs 8H , 15 , 16H

##### Type.

Panama • Chiriquí Province: W of Boquete town, Bajo Boquete community, 08°46.45'N, 082°28.03'W, about 1400 m a.s.l., evergreen montane tropical angiosperm forest, on decorticated fallen branch, 27 Jul 2019, E. Yurchenko EYu 190727-25 (***holotype***: BLS M-2668; ***isotype***: CFMR). GenBank: ITS = PP471817.

##### Etymology.

The epithet refers to the subcylindrical shape of cystidia and basidiospores.

##### Description.

Basidiomata effused, 0.15–1.5 cm in extent, soft- to moderately membranaceous, 30–85 μm thick between aculei. Hymenial surface minutely odontoid or warted, dirty white with faint cream tinge; aculei or warts conical to cylindrical, 6–8/mm, 30–55 μm high, 20–50 μm in diam. Margin slightly paler than the main surface, mould-like, 0.3–1 mm wide. Hyphal system monomitic, hyphae clamped at all septa, moderately branched, colourless, with scattered to abundant loosely attached rosette-shaped crystals. Subicular hyphae (1.7–)2–4 μm wide, thin- to slightly thick-walled; subhymenial hyphae 1.7–4.3 μm wide, thin-walled, somewhat short-celled and inflated. Cystidia smooth or variously encrusted, of several types: 1) subulate (fusoid) scattered to common, (18–)20–33 × 2.5–4.3 μm; 2) subcylindrical and cylindrical scarce, 18–32 × 2.5–4.5 μm; 3) subcapitate or capitulate rare, 12–20 × 2–3 μm. Outgrowths of hymenial surface consisting in the upper part of subulate and subcylindrical cystidia. Basidioles ovoid, short cylindrical, subclavate, 7.5–13.5 × 3.5–4.5 μm. Basidia utriform to narrowly utriform, 15–20 × 3.5–4.5 μm; sterigmata four, 2–3 × 0.7–1 μm. Basidiospores narrowly ellipsoid to subcylindrical, with adaxial side from slightly convex to slightly concave, (4.5–)5–6(–6.5) × 2.5–3.3(–3.5) μm (in holotype L = 5.5 μm, W = 2.95 μm), Q = 1.6–1.8(–2), thin-walled, smooth, colourless, Mz–, acyanophilous; apiculus minute.

**Figure 15.**
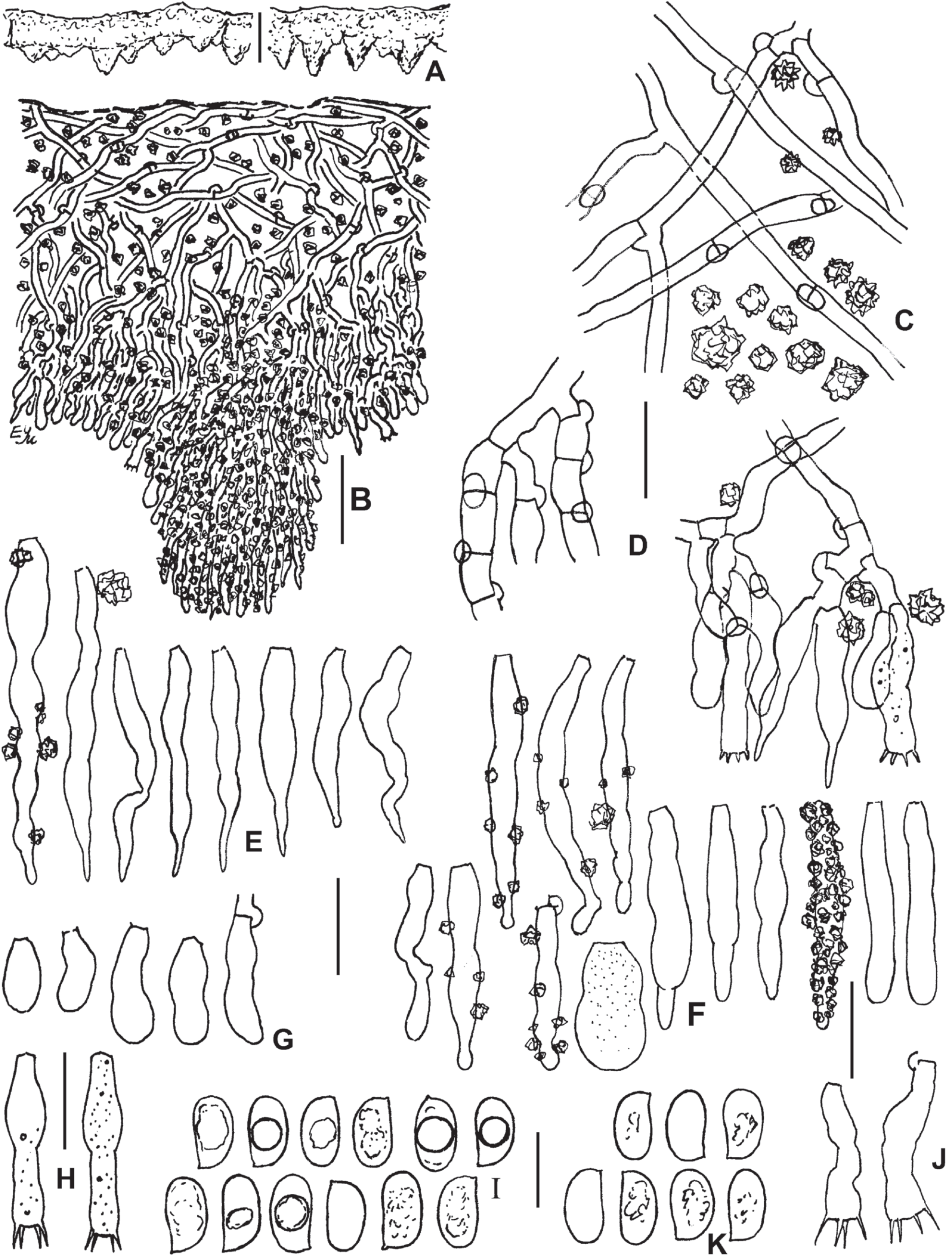
Micromorphology of *Lyomycessubcylindricus* (holotype, BLS M-2668) **A, B** vertical sections through basidioma **C** subicular hyphae **D** portions of subhymenium and hymenium **E** Subulate cystidia **F** capitulate, subcylindrical, and cylindrical cystidia **G** basidioles **H** basidia **I** basidiospores. BLS M-2992: **J** basidia **K** basidiospores. Scale bars: 100 μm (**A**); 20 μm (**B**); 10 μm (**C–H, J**); 5 μm (**I, K**).

##### Distribution.

So far, known from the western part of Panama.

##### Ecology.

The fungus grows on dead wood in evergreen montane tropical angiosperm forests.

##### Additional specimen examined

**(*paratype*).** Panama • Chiriquí Province: W of Boquete town, Bajo Boquete community, 08°46.58'N, 082°28.17'W, about 1450 m a.s.l., evergreen montane tropical angiosperm forest, bottom of canyon with a rivulet, on a fallen partly corticated woody stem, 27 Jul 2019, E. Yurchenko EYu 190727-10a (BLS M-2992). GenBank: ITS = PP471816; 28S = PP471832.

##### Notes.

The main diagnostic features of this species are minutely odontoid, dirty white basidiomata, thin-walled hyphae, the presence of subulate and subcylindrical cystidia, and subcylindrical, thin-walled basidiospores. The species is morphologically closest to *L.crustosus*. It differs from the latter by its whitish, densely and minutely odontoid hymenial surface, looser texture of basidioma, not copious subulate cystidia, and the presence of subcylindrical cystidia.

**Figure 16.**
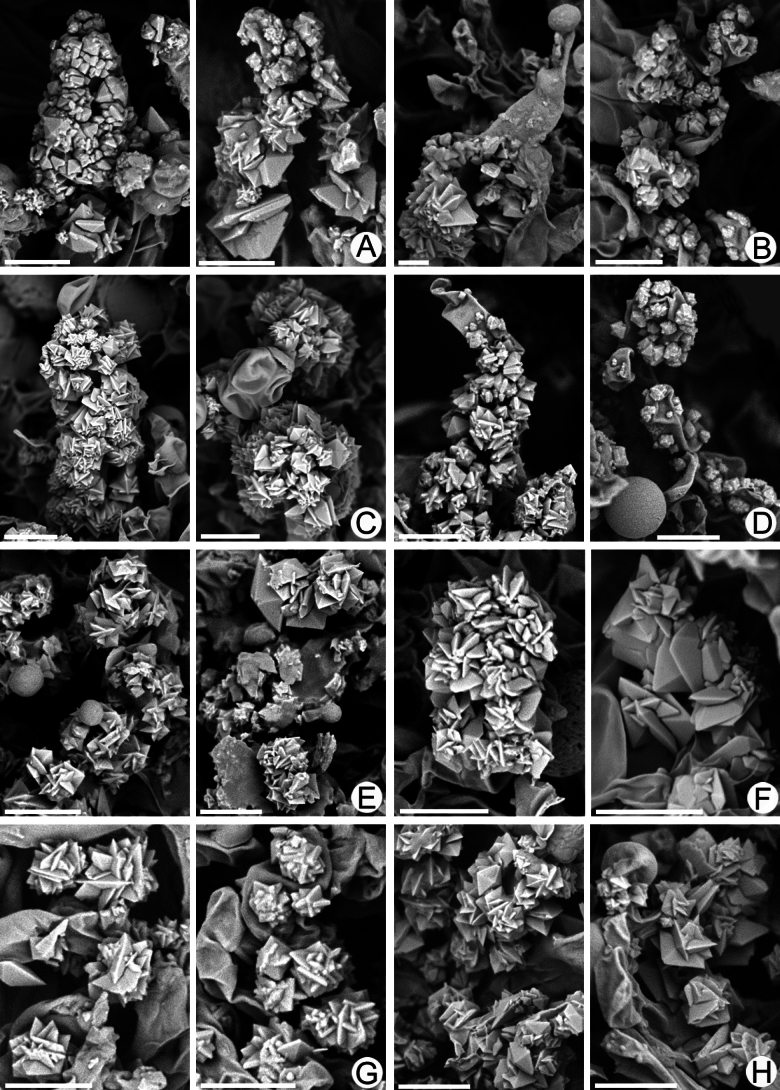
SEM of crystalline deposits on hymenial elements and hyphae in holotypes of *Lyomyces* species **A, B***L.boquetensis***C***L.granulosus***D***L.napoensis***E***L.neocrustosus***F***L.pantropicus***G***L.parvus***H***L.subcylindricus*. Scale bars: 5 μm.

### ﻿Morphology of crystalline deposits in the basidiomata of new *Lyomyces* species

Light microscopy has revealed notable crystalline incrustations on cystidia, other hymenial elements, and on hyphae in several new *Lyomyces* species. Subsequent SEM analysis showed that individual crystals are mostly of a bipyramidal shape, which is typical for calcium oxalate. However, there are differences in size and the degree of aggregation of these crystals among species (Fig. 16). In *L.boquetensis*, both small-sized and large crystals are densely aggregated; *L.granulosus* has mostly low-bipyramidal crystals, collected in compact globose structures; *L.napoensis* exhibits mostly small-sized crystals, both aggregated and dispersed; *L.pantropicus* shows both large and minute crystals present in dense aggregations. The highest similarity in incrustational morphology is observed among the species belonging to the *L.crustosus* group: *L.neocrustosus*, *L.parvus*, *L.subcylindricus*. After SEM study, we found that rosette-like structures, visible under light microscope in these three species (Figs 7, 13, 15), are not separate crystals, but aggregations of crystals (Fig. 16E, G, H). Additionally, there are irregular-shaped crystal assemblages of varying sizes in *L.neocrustosus* and *L.subcylindricus*; in *L.parvus* some crystals are solitary or loosely aggregated. Incrustations are fairly similar in *L.boquetensis* and *L.napoensis*, despite the species not being phylogenetically closely related.

### ﻿Extending the distribution range of *Lyomycesorganensis*

**Figure 17.**
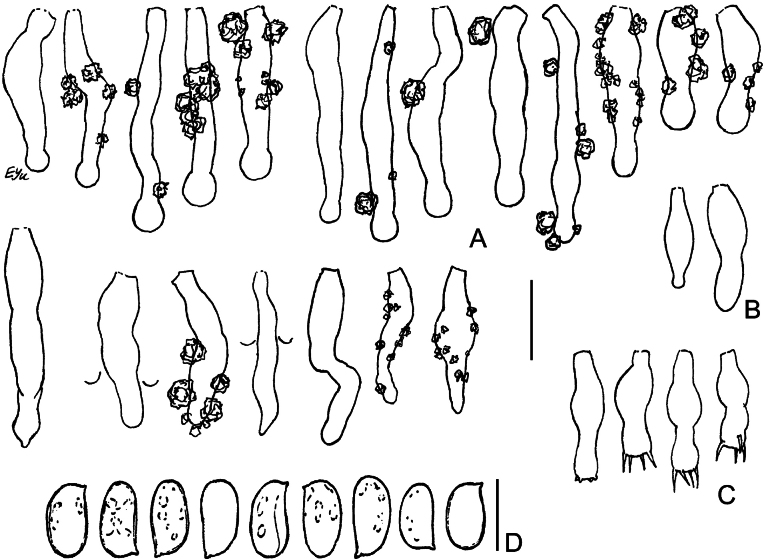
Micromorphology of *Lyomycesorganensis* (KAS-Ec180-2004) **A** cystidia **B** basidioles **C** basidia **D** basidiospores. Scale bars: 10 μm (**A–C**); 5 μm (**D**).

*Lyomycesorganensis* was previously known from only a single locality in southeastern Brazil (Yurchenko et al. 2017). The Ecuadorian specimen displays similar macromorphology; however, there are some differences in micromorphological features: the subhymenial hyphae are narrower (1.8–2.5 μm wide), the subcapitate cystidia are slightly broader (4–4.5 μm wide), the basidia are larger (14–19.5 × 4.3–4.5 μm), almost urniform, and the basidiospores are narrower, 5–6.3(–6.8) × 2.5–3(–3.3) μm, L = 5.7 μm, W = 2.85 μm (Fig. 17). Additionally, in the Ecuadorian specimen, the subhymenium is richly encrusted, while the subiculum contains only a small amount of crystalline material.

**Specimen examined.** Ecuador • Zamora Chinchipe Province: 17 km NW of Zamora, in the vicinity of Estación Científica San Francisco, between 1850 and 2450 m a.s.l., on bark and wood of dead twigs 4–7 mm in diam., 19 Jul 2004, E. Langer 180 (KAS-Ec180-2004).

## ﻿Discussion

The members of the genus *Lyomyces* are often overlooked during field studies of macrofungi due to their small size and thin (from 30–50 μm thick), non-pigmented, white or whitish basidiomata. For example, in *L.parvus*, patches with sporulating hymenium may only measure 1–3 mm in length, and their loose structure makes them difficult to observe in the field with the naked eye.

Species identification of *Lyomyces* during microscopic studies in the laboratory is also challenging due to the simple organisation of basidiomata, the accumulation of abundant crystalline deposits in them, and the poorly preserved fertile hymenium in many specimens. Furthermore, we have observed that in a tropical environment, the basidiomata of two superficially similar *Lyomyces* species can grow on the same dead wood unit or very close to each other in one locality, leading to cases of collecting two species as one mixed specimen.

Most *Lyomyces* species we identified, confirmed by rDNA ITS data, have so far been recorded exclusively in the Neotropics. *Lyomycespantropicus* can be an example of broader distribution: the high similarity in ITS sequences between specimens from the Neotropics and those from Asia suggests that the range of this new species should be expanded to the Asian continent.

The joint consideration of morphology and phylogenetic inference (Figs 1, 2) revealed several patterns in *Lyomyces*. A well-supported clade, referred to as *L.crustosus* gr. (spanning from *L.crystallina* to *L.parvus* in the phylogram—Fig. 1), consists of species possessing subulate or fusoid cystidia with usually acuminate apices, along with rosette-shaped crystals in the basidioma. *Lyomyceselaeidicola* and *L.napoensis*, both sharing basidioles and basidia with closely attached incrustations, form a cluster on the Bayesian phylogram, though without a strong support (Fig. 2). The species in a cluster, referred to as *L.sambuci* gr. (from *L.incrustatus* to *L.orientalis* on the phylogram—Fig. 1), are identified by discontinuous and flocculate to continuous and smooth basidiomata, often with numerous capitate or capitulate cystidia carrying various amounts of attached crystals.

Conversely, in a well-supported cluster *L.densiusculus*– *L.granulosus*, common features are challenging to ascertain. Species with allantoid or subcylindrical spores and the absence of subulate cystidia exhibit an isolated position on the phylogram (*L.organensis*, *L.allantosporus*). Notably, macroscopic characters show greater variability between species belonging to the same phylogenetic cluster than microscopic ones. This is particularly evident in *L.crustosus* gr., where the basidiomata vary from discontinuous, pruinose to continuous, subсeraceous.

As noted previously (Yurchenko and Wu 2014), SEM examination of crystalline deposits in *Hyphodontia* s.l. revealed differences between species, as already demonstrated by Keller (1985). In the current study, we observed that the size and arrangement of crystals in the hymenium can serve as additional morphological characters for species delimitation, although the shape of the crystals does not consistently play a defining role.

From this study, it becomes evident that the species diversity of *Lyomyces* sampled from the four countries, which collectively make up approximately 1/12^th^ of the Neotropics’ territory, is more than three times higher than the entire genus diversity found in Europe (Bernicchia and Gorjón 2010). It is essential to note that the species richness presented in this article is not a fixed number and is likely to increase with additional collection trips and more comprehensive searches of fungi in various types of ecosystems, particularly in biotopes along the altitudinal gradient.

## Supplementary Material

XML Treatment for Lyomyces boquetensis

XML Treatment for Lyomyces granulosus

XML Treatment for Lyomyces napoensis

XML Treatment for Lyomyces neocrustosus

XML Treatment for Lyomyces oleifer

XML Treatment for Lyomyces orarius

XML Treatment for Lyomyces pantropicus

XML Treatment for Lyomyces parvus

XML Treatment for Lyomyces sceptrifer

XML Treatment for Lyomyces subcylindricus

## ﻿Additional information

### Author ORCIDs

Eugene Yurchenko https://orcid.org/0000-0001-9460-3774

Ewald Langer https://orcid.org/0000-0003-3480-2936

Janett Riebesehl https://orcid.org/0000-0002-0160-4813

### Data availability

All of the data that support the findings of this study are available in the main text or Supplementary Information.

## Supplementary materials

Supplementary material 1 Alignment for the ITS dataset Data type fas Explanation note Multiple alignment. https://binary.pensoft.net/file/1147648This dataset is made available under the Open Database License (http://opendatacommons.org/licenses/odbl/1.0/). The Open Database License (ODbL) is a license agreement intended to allow users to freely share, modify, and use this Dataset while maintaining this same freedom for others, provided that the original source and author(s) are credited.Author: Janett Riebesehl

Supplementary material 2 Alignment for the combined ITS and 28S dataset Data type fas Explanation note Multiple alignment. https://binary.pensoft.net/file/1147649This dataset is made available under the Open Database License (http://opendatacommons.org/licenses/odbl/1.0/). The Open Database License (ODbL) is a license agreement intended to allow users to freely share, modify, and use this Dataset while maintaining this same freedom for others, provided that the original source and author(s) are credited.Author: Janett Riebesehl
